# Virulence Determinants and Plasmid-Mediated Colistin Resistance *mcr* Genes in Gram-Negative Bacteria Isolated From Bovine Milk

**DOI:** 10.3389/fcimb.2021.761417

**Published:** 2021-11-23

**Authors:** Yasmine H. Tartor, Rasha M. A. Gharieb, Norhan K. Abd El-Aziz, Hend M. El Damaty, Shymaa Enany, Eman Khalifa, Amira S. A. Attia, Samah S. Abdellatif, Hazem Ramadan

**Affiliations:** ^1^ Microbiology Department, Faculty of Veterinary Medicine, Zagazig University, Zagazig, Egypt; ^2^ Zoonoses Department, Faculty of Veterinary Medicine, Zagazig University, Zagazig, Egypt; ^3^ Animal Medicine Department (Infectious Diseases), Faculty of Veterinary Medicine, Zagazig University, Zagazig, Egypt; ^4^ Microbiology and Immunology Department, Faculty of Pharmacy, Suez Canal University, Ismailia, Egypt; ^5^ Biomedical Research Department, Armed Force College of Medicine, Cairo, Egypt; ^6^ Department of Microbiology, Faculty of Veterinary Medicine, Matrouh University, Marsa Matrouh, Egypt; ^7^ Veterinary Public Health Department, Faculty of Veterinary Medicine, Zagazig University, Zagazig, Egypt; ^8^ Food Control Department, Faculty of Veterinary Medicine, Zagazig University, Zagazig, Egypt; ^9^ Hygiene and Zoonoses Department, Faculty of Veterinary Medicine, Mansoura University, Mansoura, Egypt

**Keywords:** colistin, Gram-negative bacteria, mastitis, milk, *mcr*, virulence factors, multidrug-resistance

## Abstract

A major increase of bacterial resistance to colistin, a last-resort treatment for severe infections, was observed globally. Using colistin in livestock rearing is believed to be the ground of mobilized colistin resistance (*mcr*) gene circulation and is of crucial concern to public health. This study aimed to determine the frequency and virulence characteristics of colistin-resistant Gram-negative bacteria from the milk of mastitic cows and raw unpasteurized milk in Egypt. One hundred and seventeen strains belonging to *Enterobacteriaceae* (*n* = 90), *Pseudomonas aeruginosa* (*n* = 10), and *Aeromonas hydrophila* (*n* = 17) were screened for colistin resistance by antimicrobial susceptibility testing. The genetic characteristics of colistin-resistant strains were investigated for *mcr*-*1–9* genes, phylogenetic groups, and virulence genes. Moreover, we evaluated four commonly used biocides in dairy farms for teat disinfection toward colistin-resistant strains. Multidrug-resistant (MDR) and extensive drug-resistant (XDR) phenotypes were detected in 82.91% (97/117) and 3.42% (4/117) of the isolates, respectively. Of the 117 tested isolates, 61 (52.14%) were colistin resistant (MIC >2 mg/L), distributed as 24/70 (34.29%) from clinical mastitis, 10/11 (90.91%) from subclinical mastitis, and 27/36 (75%) from raw milk. Of these 61 colistin-resistant isolates, 47 (19 from clinical mastitis, 8 from subclinical mastitis, and 20 from raw milk) harbored plasmid-borne *mcr* genes. The *mcr-1* gene was identified in 31.91%, *mcr-2* in 29.79%, *mcr-3* in 34.04%, and each of *mcr-4* and *mcr-7* in 2.13% of the colistin-resistant isolates. Among these isolates, 42.55% (20/47) were *E. coli*, 21.28% (10/47) *A. hydrophila*, 19.12% (9/47) *K. pneumoniae*, and 17.02% (8/47) *P. aeruginosa*. This is the first report of *mcr*-3 and *mcr*-7 in *P. aeruginosa*. Conjugation experiments using the broth-mating technique showed successful transfer of colistin resistance to *E. coli* J53-recipient strain. Different combinations of virulence genes were observed among colistin-resistant isolates with almost all isolates harboring genes. Hydrogen peroxide has the best efficiency against all bacterial isolates even at a low concentration (10%). In conclusion, the dissemination of mobile colistin resistance *mcr* gene and its variants between MDR- and XDR-virulent Gram-negative isolates from dairy cattle confirms the spread of *mcr* genes at all levels; animals, humans, and environmental, and heralds the penetration of the last-resort antimicrobial against MDR bacteria. Consequently, a decision to ban colistin in food animals is urgently required to fight XDR and MDR bacteria.

## Introduction

*Escherichia coli*, *Klebsiella pneumoniae*, and *Pseudomonas aeruginosa* are the most common Gram-negative environmental pathogens in dairy farms. They could infect the mammary glands of dairy cows through the teat end and colonize the mammary tissue, causing mastitis (Scatamburlo et al., 2015; Gao et al., 2017). Although *Aeromonas* species are autochthonous to aquatic environments, *Aeromonas hydrophila* has been reported as a common contaminant in a diverse variety of foods such as milk and milk products (Sharma and Kumar, 2011). Contamination of milk with pathogenic Gram-negative bacteria is often derived directly from the udder excretion of infected animals or the farm environment, thus putting the consumers who consume unpasteurized milk at a high risk for developing foodborne diarrheal diseases (Garcia et al., 2019).

In Egypt, there are no regulations that control the use of antibiotics in animal husbandry for growth promotion or the prevention and treatment of bacterial diseases (WHO, 2013). The misuse of antibiotics in dairy production is a matter of concern for the effective antibiotic therapy of infected cows, food safety, and occupational exposure, and a potential threat to public health due to the selection of multidrug-resistant (MDR) bacteria and entry of antibiotic residues into bulk tank milk (Oliveira and Ruegg, 2014; Locatelli et al., 2019). The emergence of MDR and extensive drug-resistant (XDR) Gram-negative bacteria in livestock production increased the interest in the use of colistin (polymyxin E) as a last-resort antibiotic for the treatment of these pathogens (Kempf et al., 2016). Although colistin was prohibited in developed countries, it is still used in animal husbandry in Egyptian dairy farms (Lima Barbieri et al., 2017; Dandachi et al., 2019). Hence, the extensive use of colistin increases colistin-resistant Gram-negative bacteria. Also, it imposes a major public health concern (Lima Barbieri et al., 2017) due to the dissemination of colistin resistance genes (*mcr*) through mobilized plasmids among animal strains and subsequently transmitted to humans through the food chain or direct contact (McEachran et al., 2015; García et al., 2018; Hmede and Kassem, 2019). Nine plasmid-borne *mcr*-family genes (*mcr-1*–*mcr*-9) have been reported in over 40 countries from different continents across the globe; most of them were detected in several enterobacteria, including *E. coli* and *K. pneumoniae* (Luo et al., 2020). The *mcr*-*1* gene has been detected in most continents, while other genes have only been found in a few countries (Ling et al., 2020). The *mcr-1* gene has been reported previously in colistin-resistant *E. coli* isolated from raw milk (Coton et al., 2012; Hassen et al., 2019), mastitis bovine milk (Filioussis et al., 2019; Liu G. et al., 2020), and cheese (Coton et al., 2012; Hammad et al., 2019). A higher frequency of *mcr-1* compared with other *mcr* genes has been reported in colistin-resistant *E. coli* (98.9%), *K. pneumoniae* (100%), and *P. aeruginosa* (100%) recovered from animal and human sources (Javed et al., 2020). The implementation of pre- and post-milking teat disinfection is a critical effective means of reducing the incidence of clinical and subclinical mastitis as well as new intramammary infections caused by Gram-negative environmental pathogens in dairy farms such as *E. coli*, *K. pneumoniae*, and *P. aeruginosa* (Tiwari, 2013; Kamal and Bayoumi, 2015; Böhm et al., 2017; Rowe et al., 2018). Choosing the ideal disinfectant with an accurate concentration is vital to control resistant bacteria in dairy farms. The resistant bacteria can survive at concentrations many folds below the minimum inhibitory concentration (MIC) of the biocidal agents (Hughes and Andersson, 2012). Notably, Gram-negative bacteria have a risk for promoting antibiotic resistance after exposure to sublethal concentrations of some disinfectants such as chlorhexidine (Kampf, 2018).

Most putative virulence determinants contributing to the pathogenicity of *E. coli*, *K. pneumoniae*, *Aeromonas* species, and *P. aeruginosa* are chromosomally encoded. However, previous studies have indicated the occurrence of some virulence factors on plasmids of *E. coli* (Rodriguez-Siek et al., 2005; Johnson et al., 2006; Mellata et al., 2010; Cyoia et al., 2015), *K. pneumoniae* (Dolejska et al., 2013; Chen et al., 2020), *Aeromonas* species (Brown et al., 1997), and *P. aeruginosa* (Morales-Espinosa et al., 2012). *E. coli* strains possess virulence genes that play a significant role in the survival and pathogenesis of the strain in the host through bacterial adhesion (*ipfA*), hemolysis (*hlyF*), iron acquisition (*iroN* and *ireA*), and increased serum survival and resistance to phagocytosis (*iss* and *traT*) (Sarowska et al., 2019; Adzitey et al., 2020). *K. pneumoniae* hypervirulent strains possess different genes that determine virulence and severity of infection in the host. These virulence genes include *entB* (enterobactin biosynthesis gene), *allS* (associated with allantoin metabolism), *ybtS* and *irp* (yersiniabactin biosynthesis), and *mrkD* and *fimH* (fimbrial adhesin that mediates binding to the extracellular matrix to form the biofilm) (Rosen et al., 2008; Wasfi et al., 2016; Ye et al., 2016; Russo and Marr, 2019). The most important *A. hydrophila* virulence factors include hemolysin A (*hlyA*), aerolysin (*aer*), cytotonic heat-stable enterotoxin (*ast*), cytotoxic enterotoxin (*act*), and lipases (*lip*) (Furmanek-Blaszk, 2014; Rather et al., 2014). *P. aeruginosa* produces extracellular products that contribute to its pathogenicity, such as protein exotoxin A (*toxA*), proteases (*lasB*), type III secretion system exoenzymes (*exoU* and *exoS*) (Casilag et al., 2016; Fadhil et al., 2016), and genes responsible for pyocyanin production (*phzM*) (Nowroozi et al., 2012). Genes encoding virulence and MDR/XDR are often found with *mcr* genes on plasmids in environmental isolates (Anyanwu et al., 2020). There are limited reports that studied the occurrence of plasmid-encoded virulence and colistin resistance genes (*mcr*) among colistin-resistant Gram-negative bacteria isolated from cow’s milk in our geographic region. Hence, the current study aimed to: (1) determine the frequency of colistin-resistant Gram-negative bacteria from the milk of mastitic cows as well as raw unpasteurized milk; (2) screen for *mcr* resistance determinants in bacterial isolates and their virulence characteristics; (3) evaluate four frequently used biocides in dairy farms for teat disinfection toward colistin-resistant isolates.

## Materials and Methods

### Gram-Negative Bacterial Strains

A total of 117 Gram-negative bacterial strains belonging to *Enterobacteriaceae* (*n* = 90), *P. aeruginosa* (*n* = 10), and *A. hydrophila* (*n* = 17) were included in this study. The bacterial isolates were recovered previously from milk samples of dairy cows showing mastitis as well as bulk tank raw milk during 2018–2020. Gram-negative isolates were selected based on the provided data from the Microbiology Department, Faculty of Veterinary Medicine, Zagazig University, Egypt. *Enterobacteriaceae* strains included non-O_157_ *E. coli* (*n* = 30), O_157_ *E. coli* (*n* = 12), *K. pneumoniae* (*n* = 18), *Enterobacter* species (*n* = 10), and *Citrobacter* species (*n* = 20). The isolated bacteria were sourced from different farms. All the isolates were independent and were not clonally duplicated from the same source. The presumptive bacterial isolates were confirmed adopting the standard microbiological procedures (Quinn et al., 1994). In brief, MacConkey’s, eosin methylene blue (Oxoid, Cambridge, UK) and HiCrome klebsiella-selective agar media (Himedia, Mumbai, India) were used for the cultivation of *Enterobacteriaceae* strains. *Aeromonas* species were grown on Rimler-Shotts medium (HiMedia, India) with a novobiocin (Oxoid, UK) supplement, while *P. aeruginosa* strains were cultivated on Pseudomonas cetrimide agar (Oxoid, UK). Serotyping of *E. coli* was applied using diagnostic polyvalent and monovalent O and H antisera (Denka Seiken Co., Tokyo, Japan). Genomic DNA was extracted from fresh bacterial cultures using the QIAamp DNA Mini kit (Qiagen, Hilden, Germany) following the manufacturer’s instructions. Polymerase chain reaction (PCR)-based confirmation of Gram-negative bacteria was applied using genus- and species-specific primer sets depicted in **Supplementary Table S1**.

### Antimicrobial Susceptibility Testing

The antimicrobial susceptibilities of Gram-negative bacterial strains (*n* = 117) against a panel of 24 commonly used antimicrobial agents were determined using the Kirby-Bauer disk diffusion assay following the Clinical and Laboratory Standards Institute guidelines and interpretative criteria (CLSI, 2019). The tested antimicrobial discs (Oxoid, Cambridge, UK) were ampicillin (10 µg), amoxicillin-clavulanic acid (30 µg), piperacillin-tazobactam (40 µg), cefazolin (30 µg), cephalothin (30 µg), cefoxitin (30 µg), ceftriaxone (30 µg), cefotaxime (30 µg), ceftazidime (30 µg), cefepime (30 µg), imipenem (10 µg), nalidixic acid (30 µg), ciprofloxacin (5 µg), levofloxacin (5 µg), gentamicin (10 µg), tobramycin (10 µg), amikacin (30 µg), trimethoprim-sulphamethoxazole (25 µg), chloramphenicol (30 µg), tetracycline (30 µg), aztreonam (30 µg), tigecycline (15 µg), fosfomycin (50 µg), and colistin (25 µg). The minimum inhibitory concentration (MIC) of colistin (Sigma-Aldrich, Seelze, Germany) was determined against Gram-negative bacterial strains by broth microdilution technique following the relevant CLSI document (CLSI, 2019).

The multiple antibiotic resistance (MAR) indices were evaluated as documented elsewhere (Tambekar et al., 2006), while the MDR and XDR phenotypes were reported according to Magiorakos et al. (2012). *E. coli* ATCC 25922, *P. aeruginosa* ATCC 27853, and *A. hydrophila* ATCC 7966 were used as quality control strains.

### Detection of Plasmid-Mediated Colistin Resistance Genes

Plasmid DNAs were extracted from colistin-resistant Gram-negative bacterial strains (*E. coli*, *K. pneumoniae*, *P. aeruginosa*, and *A. hydrophila*) using Plasmid DNA Miniprep Kits (Thermo Fisher Scientific, Waltham, MA, USA) following the manufacturer’s instructions. PCR amplifications of colistin resistance genes (*mcr-1* to *mcr-9*) from phenotypic colistin-resistant Gram-negative bacteria isolates were performed using oligonucleotide primer sequences and their annealing temperatures listed in **Supplementary Table S1**. Conventional PCRs were performed using a programmable 2720 thermal cycler (Applied Biosystem, Waltham, MA, USA) in a total reaction volume of 25 μl containing 12.5 μL of EmeraldAmp Max PCR Master Mix (Takara, Japan), 1 μl of each primer (20 pmol) (Sigma-Aldrich, Co., St. Louis, MO, USA), 5 μl of template DNA, and 5.5 μl of nuclease-free water. The amplified products were visualized after 30 min of electrophoresis on a 2% agarose gel containing ethidium bromide.

### DNA Sequencing and Phylogenetic Analysis of *mcr* Genes

DNA sequencing of the PCR products was applied to validate their genetic identity and to verify any mutations in the *mcr* genes. The amplified DNA fragments were purified with the QIAquick PCR purification kit (Qiagen, Courtaboeuf, France). Sanger sequencing was performed in both directions using Bigdye Terminator V3.1 cycle sequencing kit (Perkin-Elmer, Inc. Waltham, MA, USA) in an applied Biosystems 3130 genetic analyzer (Hitachi, Tokyo, Japan). The nucleotide sequences were compared with those available in the National Center for Biotechnology Information (NCBI; www.ncbi.nlm.nih.gov). Nucleotide and deduced protein sequences were analyzed using the MEGA7 program (Kumar et al., 2016). To investigate the genetic relatedness and the evolutionary distance among Gram-negative strains harboring the *mcr* genes, a phylogenetic tree was constructed using the neighbor-joining (maximum composite likelihood) method. DNA sequences generated in the study were deposited into the GenBank database with accession numbers MW811398-MW811434 and MZ648218-MZ648227.

### Conjugation Assay

The transmissibility of colistin-resistant *mcr* genes between donors (*mcr*-positive isolates) and the recipient bacteria (*E. coli* J53; Na azide-resistant) was evaluated by the conjugation assays using the broth mating technique (Wang et al., 2003). An equal ratio (1:1) of donor and recipient cells (0.5 ml of each) in a logarithmic phase were added to 4 ml of sterile Luria-Bertani (LB) broth (Sigma-Aldrich, USA) then incubated overnight at 37°C without shaking. The mating mixture was serially diluted then the transconjugants were cultured on LB agar (Sigma-Aldrich, USA) supplemented with colistin (2 mg/L) or Na azide (100 mg/L). MICs for the donors, recipients, and transconjugants were determined by the broth microdilution method following the CLSI guidelines (CLSI, 2019). Potential transconjugants were examined for the existence of *mcr* genes using PCR assays. The conjugation efficiency was calculated as the number of transconjugants per donor as previously documented (Wang et al., 2003).

### PCR Amplification of Virulence-Associated Genes

PCR detection of virulence-related genes of *E. coli* (*hlyF, ireA, iroN, iss, lpfA,* and *traT*), *K. pneumoniae* (*entB, alls, mrkD, fimH, ybtS,* and *irp1*), *P. aeruginosa* (*exoU, exoS, lasB, toxA,* and *phzM*), and *A. hydrophila* (*hlyA, aer, lip, ast,* and *act*) was performed in conventional PCR assays using the oligonucleotide primer sequences presented in **Supplementary Table S1**.

### Disinfectant Susceptibility Testing

Gram-negative bacterial strains (three of each species exhibiting colistin resistance) were evaluated against four commonly used biocides in dairy farms for teat disinfection, namely chlorhexidine gluconate (0.5% in alcohol 70%; Pfizer, Manhattan, NY, USA), hydrogen peroxide (H_2_O_2_; 6%, *w*/*v*), iodine (0.5%, *w*/*v*), and alcohol (ethanol; 70% *v*/*v*) (El Nasr Pharmaceutical Chemicals Co., Cairo, Egypt). The disinfectants were diluted (10–90%) using sterile distilled water, in which the original concentrations were considered stock solutions. The agar well diffusion method was applied to evaluate their effectiveness against the suspected strains of equivalent turbidity to that of 0.5 McFarland standards. Diameters of inhibition zones from the well edge to the inhibition front equal to or larger than 15 mm were regarded as susceptible (Wanja et al., 2020). The MICs of teat disinfectants against bacterial strains were determined as described before (Baron and Feingold, 1990).

### Bioinformatics and Data Analysis

Statistical analyses were performed using the software SAS (SAS Institute Inc., 2012). Results of conjugation efficiency of Gram-negative bacteria were graphed by a boxplot through GraphPad Software (version 8.0.1, GraphPad Software Inc., La Jolla, CA, USA). Resistance phenotypes and frequencies of virulence genes among isolates were compared using the *Chi-*squared (*χ* ^2^) test. *P* < 0.05 was considered significant. A heatmap with hierarchical clustering was generated to visualize the overall distribution of *mcr* variants and antimicrobial resistance phenotypes in colistin-resistant isolates using the “pheatmap” package in R software (version 3.4.2) (Kolde, 2019). To determine the shared *mcr* variants among colistin-resistant isolates from different sources, a Venn diagram was generated using the *Venny 1.0* tool, https://bioinfogp.cnb.csic.es/tools/venny/index.html. Spearman correlations were used to provide an estimate for the association among various variables (resistance phenotypes, colistin resistance, and virulence genes). The correlation coefficients and their *p*-values were visualized using a correlation plot. The correlation analyses and visualization were done using R packages *rcorr* (https://hbiostat.org/R/Hmisc/) and *corrplot* (https://github.com/taiyun/corrplot). Genes or phenotypes that were present or absent in all analyzed subjects were not considered in these analyses.

## Results

### Resistance Phenotypes of Gram-Negative Bacteria Isolated From Mastitis and Raw Milk

Overall, the MDR phenotype was determined for 70 *Enterobacteriaceae* isolates including 37/42 (88.1%) *E. coli* (27/30 were non-O_157_ and 10/12 were O_157_ *E. coli*), 18/18 K*. pneumoniae* (100%), 10/10 *Enterobacter* species (100%), and 5/20 *Citrobacter* species (25%). All *P. aeruginosa* and *A. hydrophila* isolates were MDR with MAR indices ranged from 0.33 to 0.79. Three *P. aeruginosa* and one *E. coli* (O111:H4) exhibited an XDR phenotype as being resistant to at least one antibiotic of all tested antimicrobial classes but remained susceptible to two classes (carbapenems and monobactams in *P. aeruginosa*, carbapenems and glycylcyclines in *E. coli*).

*E. coli* isolates showed high resistance to ampicillin, cephalothin, cefazolin, ceftazidime (100% each), cefoxitin (97.62%), cefotaxime (95.23%), and amoxicillin-clavulanic acid (90.47%). In addition, over 50% of isolates were resistant to piperacillin-tazobactam (71.43%), colistin, and fosfomycin (57.14%, each).

All *K. pneumoniae* isolates were resistant to ampicillin, amoxicillin-clavulanic acid, fosfomycin, cefoxitin, cefazolin, and cephalothin. Meanwhile, 94.4% of the isolates were resistant to both ceftazidime and piperacillin-tazobactam, 88.89% were resistant to tigecycline, 77.78% to ceftriaxone, cefotaxime, and colistin, and 55.56% were resistant to sulfamethoxazole-trimethoprim.

*A. hydrophila* isolates were sensitive to amikacin, imipenem, ciprofloxacin, and levofloxacin (100% each). However, the highest resistance was for the tetracycline and cephalosporins groups (ceftriaxone, cefotaxime, ceftazidime, cefoxitin, and cephalothin (100% each)) followed by colistin (88.24%), nalidixic acid (76.47%), sulfamethoxazole-trimethoprim (70.59%), and cefazolin (58.8%).

*P. aeruginosa* isolates exhibited resistance to sulfamethoxazole-trimethoprim, amoxicillin-clavulanic acid, fosfomycin, cefoxitin, cefazolin, and cephalothin (100% each); tetracycline, chloramphenicol, and ampicillin (90% each); colistin and nalidixic acid (80% each); and ceftazidime, ciprofloxacin, and levofloxacin (60% each). However, the most active antimicrobial agents against *P. aeruginosa* isolates were imipenem (100%), aztreonam (90%), piperacillin-tazobactam, and ceftriaxone (80% each).

Cephalosporins (cefotaxime, ceftazidime, cefoxitin, cefazolin, and cephalothin), ampicillin, and amoxicillin-clavulanic acid were the least active antimicrobials against both *Enterobacter* and *Citrobacter* species as well as cefepime, sulfamethoxazole-trimethoprim, and tetracycline in *Enterobacter* species (100% resistance rate). *Citrobacter* species were completely sensitive to both sulfamethoxazole-trimethoprim and tetracycline. Meanwhile, both species were highly sensitive to imipenem, gentamicin, tobramycin, amikacin, ciprofloxacin, levofloxacin, colistin, piperacillin-tazobactam, and tigecycline (100% each).

### Trends in Bacterial Resistance and MAR Indices

The overall resistance rates of Gram-negative isolates from clinical mastitis, subclinical mastitis, and raw milk to each tested antimicrobial were analyzed using *χ* ^2^ test. As revealed in **Table 1**, a significant (*p* **<** 0.05) difference in resistance rates of isolates from clinical, subclinical mastitis, and raw milk was observed against ampicillin, cefazolin, ceftriaxone, nalidixic acid, tobramycin, fosfomycin, colistin, and tetracycline. All isolates from different sources were highly resistant to the first and second generations of cephalosporins especially cephalothin (100%). Furthermore, the MAR index of each antimicrobial was calculated to understand the level of antibiotic use. High MAR index (0.04) of ampicillin, amoxicillin-clavulanic acid, and the first and second generations of cephalosporins was observed. This implies the excessive use of these antimicrobials in the veterinary sector and human medicine. Meanwhile, imipenem achieved a lower MAR index.

**Table 1:** Resistance rates of 117 Gram-negative bacteria from milk samples against the tested antimicrobial agents and the overall multiple antibiotic resistance (MAR) index.

AMA	Clinical mastitis					Subclinical mastitis			Raw milk			MAR index^a^	*p-*value^*^
	*E. coli* (*n* = 25)	*K. pneumoniae* (*n* = 8)	*Enterobacter* species (*n* = 10)	*Citrobacter* species (*n* = 20)	*P. aeuriginosa* (*n* = 7)	*E. coli* (*n* = 5)	*K. pneumoniae* (*n* = 3)	*P. aeuriginosa* (*n* = 3)	*E. coli* (*n* = 12)	*K. pneumoniae* (*n* = 7)	*A. hydrophila* (*n* = 17)		
AM	25 (100.00)	8 (100.00)	10 (100.00)	20 (100.00)	6 (85.71)	5 (100.00)	3 (100.00)	3 (100.00)	12 (100.00)	7 (100.00)	9 (52.94)	0.04	0.0004
AMC	25 (100.00)	8 (100.00)	10(100.00)	20 (100.00)	7 (100.00)	5 (100.00)	3 (100.00)	3 (100.00)	8 (66.67)	7 (100.00)	8 (47.06)	0.04	0.0000007
CEF	25 (100.00)	8 (100.00)	10 (100.00)	20 (100.00)	7 (100.00)	5 (100.00)	3 (100.00)	3 (100.00)	12 (100.00)	7 (100.00)	17 (100.00)	0.04	NA
CFZ	25 (100.00)	8 (100.00)	10 (100.00)	20 (100.00)	7 (100.00)	5 (100.00)	3 (100.00)	3 (100.00)	12 (100.00)	7 (100.00)	10 (58.82)	0.04	0.0002
FOX	24 (96.00)	8 (100.00)	10 (100.00)	20 (100.00)	7 (100.00)	5 (100.00)	3 (100.00)	3 (100.00)	12 (100.00)	7 (100.00)	17 (100.00)	0.04	0.7128
CAZ	25 (100.00)	8 (100.00)	10 (100.00)	20 (100.00)	4 (57.14)	5 (100.00)	3 (100.00)	2 (66.67)	12 (100.00)	6 (85.71)	17 (100.00)	0.04	0.6633
CTX	23 (92.00)	5 (62.50)	10 (100.00)	20 (100.00)	1 (14.28)	5 (100.00)	3 (100.00)	1 (33.33)	12 (100.00)	6 (85.71)	17 (100.00)	0.04	0.1211
CRO	6 (24.00)	6 (75.00)	5 (50.00)	15 (75.00)	2 (28.57)	0 (00.00)	3 (100.00)	0 (00.00)	6 (50.00)	6 (85.71)	17 (100.00)	0.02	0.00088
FEB	9 (36.00)	2 (25.00)	10 (100.00)	0 (00.00)	1 (14.28)	2 (40.00)	1 (33.33)	1 (33.33)	5 (41.67)	4 (57.14)	4 (23.53)	0.01	0.8673
IPM	0 (00.00)	1(12.50)	0 (00.00)	0 (00.00)	0 (00.00)	0 (00.00)	0 (00.00)	0 (00.00)	0 (00.00)	0 (00.00)	0 (00.00)	0.00	0.7128
NA	5 (20.00)	0 (00.00)	0 (00.00)	5 (25.00)	5 (71.43)	1 (20.00)	0 (00.00)	3 (100.00)	5 (41.67)	1 (14.28)	13 (76.47)	0.01	0.00466
CIP	4 (16.00)	0 (00.00)	0 (00.00)	0 (00.00)	4 (57.14)	0 (00.00)	0 (00.00)	2 (66.67)	4 (33.33)	2 (28.57)	0 (00.00)	0.006	0.7614
LVX	4 (16.00)	0 (00.00)	0 (00.00)	0 (00.00)	4 (57.14)	0 (00.00)	0 (00.00)	2 (66.67)	4 (33.33)	0 (00.00)	0 (00.00)	0.005	0.799
TOB	1 (4.00)	0 (00.00)	0 (00.00)	0 (00.00)	3 (42.86)	2 (40.00)	2 (66.67)	1 (33.33)	0 (00.00)	0 (00.00)	4 (23.53)	0.005	0.0005
AK	0 (00.00)	0 (00.00)	0 (00.00)	0 (00.00)	3 (42.86)	0 (00.00)	0 (00.00)	1 (33.33)	0 (00.00)	0 (00.00)	0 (00.00)	0.001	0.2763
CN	3 (12.00)	0 (00.00)	0 (00.00)	0 (00.00)	3 (42.86)	0 (00.00)	0 (00.00)	1 (33.33)	3 (25.00)	0 (00.00)	4 (23.53)	0.005	0.2512
SXT	10 (40.00)	6 (75.00)	10 (100.00)	0 (00.00)	7 (100.00)	0 (00.00)	1 (33.33)	3 (100.00)	4 (33.33)	3 (42.86)	12 (70.59)	0.02	0.623
TGC	11 (44.00)	8 (100.00)	0 (00.00)	0 (00.00)	3 (42.86)	1 (20.00)	3 (100.00)	1 (33.33)	2 (16.67)	5 (71.43)	6 (35.29)	0.01	0.632
ATM	5 (20.00)	1 (12.50)	0 (00.00)	0 (00.00)	0 (00.00)	0 (00.00)	0 (00.00)	0 (00.00)	0 (00.00)	0 (00.00)	8 (47.06)	0.005	0.068
TPZ	21(84.00)	8 (100.00)	0 (00.00)	0 (00.00)	0 (00.00)	3 (60.00)	3 (100.00)	0 (00.00)	5 (41.67)	6 (85.71)	1 (5.88)	0.02	0.429
CHL	5 (20.00)	2 (25.00)	0 (00.00)	0 (00.00)	6 (85.71)	0 (00.00)	0 (00.00)	3 (100.00)	5 (41.67)	1 (14.28)	7 (41.17)	0.01	0.1378
FOF	15 (60.00)	8 (100.00)	0 (00.00)	0 (00.00)	7 (100.00)	5 (100.00)	3 (100.00)	3 (100.00)	3 (25.00)	7 (100.00)	2 (11.76)	0.02	0.0001
CST	15 (60.00)	4 (50.00)	0 (00.00)	0 (00.00)	5 (71.43)	4 (80.00)	3 (100.00)	3 (100.00)	5 (41.67)	7 (100.00)	15 (88.23)	0.02	0.00001
TET	9 (36.00)	4 (50.00)	10 (100.00)	0 (00.00)	6 (85.71)	2 (40.00)	1 (33.33)	3 (100.00)	6 (50.00)	2 (28.57)	17 (100.00)	0.02	0.0232

AMA, antimicrobial agent; AM, ampicillin; AMC, amoxicillin-clavulanic acid; CEF, cephalothin; CFZ, cefazolin; FOX, cefoxitin; CAZ, ceftazidime; CTX, cefotaxime; CRO, ceftriaxone; FEB, cefepime; IPM, imipenem; NA, nalidixic acid; CIP, ciprofloxacin; LVX, levofloxacin; TOB, tobramycin; AK, amikacin; CN, gentamicin; SXT, sulfamethoxazole-trimethoprim; TGC, tigecycline; ATM, aztreonam; TPZ, piperacillin-tazobactam; CHL, chloramphenicol; FOF, fosfomycin; CST, colistin; TET, tetracycline; MAR, multiple antibiotic resistance; NA, not applicable.

a MAR index for each antimicrobial = total number of resistance scored/total number of antimicrobials tested × total number of isolates (Tambekar et al., 2006).

^*^Trends in bacterial resistance to the tested antimicrobials using χ^2^ test; p < 0.05 was considered significant.

### Frequency of Colistin Resistance *mcr* Genes in MDR and XDR Gram-Negative Bacteria

In total, 61/117 tested isolates (52.14%), 24/70 (34.29%) from clinical mastitis, 10/11 (90.91%) from subclinical mastitis, and 27/36 (75%) from raw milk were colistin resistant, showing colistin MICs >2 mg/L. Among them, 19 (27.14%), 8 (72.73%), and 20 (55.56%) isolates from clinical, subclinical mastitis, and raw milk, respectively, were positive for plasmid-borne *mcr* genes.

**Figure 1:**
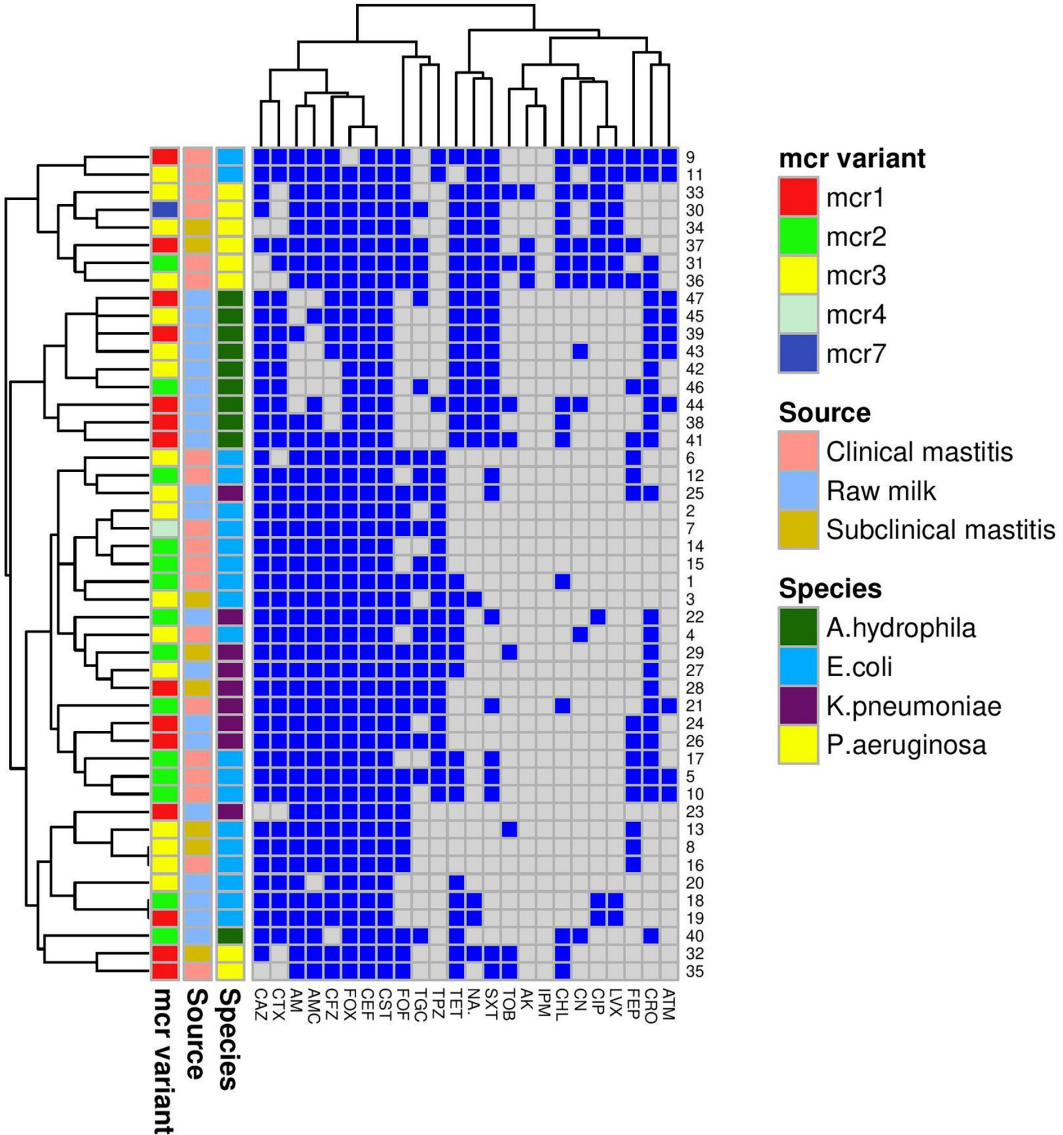
A heatmap supported by a dendrogram for colistin-resistant Gram-negative isolates (*n* = 47) from bovine mastitis and raw milk showing their antimicrobial resistance phenotypes and the distribution of *mcr* genes. *Mcr* variant, source, and species are color-coded feature categories. AM, ampicillin; AMC, amoxicillin-clavulanic acid; TPZ, piperacillin-tazobactam; CFZ, cefazolin; CEF, cephalothin; FOX, cefoxitin; CAZ, ceftazidime; CTX, cefotaxime; CRO, ceftriaxone; FEB, cefepime; IPM, imipenem; CN, gentamicin; TOB, tobramycin; AK, amikacin; NA, nalidixic acid; CIP, ciprofloxacin; LVX, levofloxacin; CST, colistin; TET, tetracycline; TGC, tigecycline; CHL, chloramphenicol; SXT, sulfamethoxazole-trimethoprim; ATM, aztreonam; FOF, fosfomycin.

From the PCR and DNA sequencing results, the *mcr-1* gene was identified in 31.91% (13 MDR and two XDR isolates), *mcr-2* in 29.79% (13 MDR and one XDR), *mcr-3* in 34.04% (15 MDR and one XDR), and each of *mcr-4* and *mcr-7* in 2.13% of the colistin-resistant Gram-negative isolates (**Table 2**). Of these isolates, 42.55% (20/47) were *E. coli*, 21.28% (10/47) *A. hydrophila*, 19.12% (9/47) *K. pneumoniae*, and 17.02% (8/47) *P. aeruginosa* (**Figure 1**).

**Table 2:** Resistance phenotypes, virulence genes profiles, and plasmid-mediated *mcr* genes of 47 Gram-negative bacteria recovered from milk samples.

Isolate No.	Species	Source	Antimicrobial resistance patterns	MAR index	MIC (mg/L)	*mcr* gene	Accession No.	Virulence gene
1	*E. coli* O111:H4	Clinical mastitis	CFZ, FOX, AM, AMC, TPZ, CEF, CAZ, FOF, CTX, TET,CHL, TGC, CST	0.54	128	*mcr-2*	MW811407	*hlyF, ireA, iss, lpfA*
2	O26:H11	Raw milk	CFZ, FOX, AM, AMC, TPZ, CEF, CAZ, FOF, CTX, CST	0.42	8	*mcr-3*	MW811419	*hlyF, ireA, iroN, lpfA, traT*
3	O111:H4	Subclinical mastitis	CFZ, FOX, AM, AMC, TET, TPZ, CEF, CAZ, CST, FOF, CTX, NA	0.5	32	*mcr-3*	MW811420	*hlyF, iroN, lpfA*
4	O111:H4	Clinical mastitis	CFZ, FOX, AM, AMC, TET, TPZ, CEF, CAZ, CTX, TGC, CST, CN, CRO	0.54	64	*mcr-3*	MW811421	*ireA, iroN, iss, lpfA*
5	O114:H21	Clinical mastitis	CFZ, CRO, FOX, SXT, AM, AMC, TPZ, CEF, CAZ, TGC, CST, TET, ATM, FOF, FEP, CTX	0.67	128	*mcr-2*	MW811408	*hlyF, ireA, iroN, lpfA, traT*
6	O114:H21	Clinical mastitis	CFZ, FEP, FOX, AM, AMC, TPZ, CEF, CAZ, FOF, TGC, CST	0.46	64	*mcr-3*	MW811422	*hlyF, iroN, iss, lpfA, traT*
7	O111:H4	Clinical mastitis	CFZ, FOX, AM, AMC, TPZ, CEF, CAZ, CTX, TGC, CST, FOF	0.46	16	*mcr-4*	MW811433	*hlyF, iroN, iss, lpfA, traT*
8	O114:H4	Subclinical mastitis	CFZ, FOX, AM, AMC, CEF, CAZ, CTX, FEP, FOF, CST	0.42	16	*mcr-3*	MW811427	*hlyF, iroN, lpfA*
**9**	O111:H4	Clinical mastitis	CN, CFZ, TPZ, CEF, CRO, CAZ, CTX, FEP, NA, CIP, LVX, SXT, ATM, AM, AMC, CHL, TE, FOF, CST	0.79	16	*mcr-1*	MW811398	*hlyF, ireA, iroN, iss, lpfA, traT*
10	O114:H21	Clinical mastitis	CFZ, TPZ, CEF, CAZ, CTX, CRO, FEP, FOX, SXT, AM, ATM, AMC, TET, CST, FOF	0.63	16	*mcr-2*	MW811409	*hlyF, ireA, iroN, lpfA, traT*
11	O111:H4	Clinical mastitis	CFZ, CAZ, CEF, CRO, FEP, FOX, CTX, NA, CIP, LVX, SXT, ATM, AM, TPZ, AMC, CHL, CST, FOF	0.75	64	*mcr-3*	MW811423	*hlyF, ireA, iroN, iss, lpfA*
12	O26:H11	Clinical mastitis	CFZ, CEF, FEP, FOX, CAZ, CTX, SXT, TGC, AM, TPZ, AMC, CST	0.5	8	*mcr-2*	MW811410	*hlyF, iroN, iss, lpfA, traT*
13	O111:H4	Subclinical mastitis	CFZ, CEF, TOB, FOX, CAZ, CTX, FEP, AM, AMC, FOF, CST	0.46	128	*mcr-3*	MW811424	*ireA, iroN, iss, lpfA, traT*
14	O26:H11	Clinical mastitis	CFZ, CEF, FOX, CAZ, CTX, AM, TPZ, AMC, CST	0.38	16	*mcr-2*	MW811411	*hlyF, ireA, iroN, iss, lpfA, traT*
15	O146:H-	Clinical mastitis	CFZ, CEF, FOX, CAZ, CTX, TGC, TPZ, AM, AMC, CST	0.42	16	*mcr-2*	MW811412	*hlyF, iroN, iss, lpfA*
16	O146:H-	Clinical mastitis	CFZ, CEF, FOX, CAZ, CTX, FEP, ATM, AMC, FOF, CST	0.42	32	*mcr-3*	MW811425	*hlyF, ireA, iss, lpfA, traT*
17	O157:H7	Clinical mastitis	CFZ, CEF, FOX, CAZ, CTX, CRO, FEP, SXT, TPZ, AM, AMC, CST, TET	0.54	>128	*mcr-2*	MW811413	*hlyF, ireA, iss, lpfA, traT*
18	O157:H7	Raw milk	CFZ, CEF, FOX, CAZ, CTX, NA, CIP, LVX, AM, AMC, CST, TET	0.5	64	*mcr-2*	MW811414	*ireA, iroN, iss, lpfA, traT*
19	O157:H7	Raw milk	CFZ, CEF, FOX, CAZ, CTX, NA, CIP, LVX, AM, AMC, CST, TET	0.5	>128	*mcr-1*	MW811399	*hlyF, iroN, iss, lpfA, traT*
20	O157:H7	Raw milk	CFZ, CEF, FOX, CAZ, CTX, AM, CST, TET	0.33	128	*mcr-3*	MW811426	*hlyF, ireA, iroN, iss, lpfA*
21	*K. pneumoniae*	Raw milk	CFZ, CEF, FOX, CAZ, CTX, CRO, SXT, TGC, ATM, TPZ, AM, AMC, CHL, FOF, CST	0.63	8	*mcr-2*	MW811415	*entB, alls, mrkD, fimH, ybtS*
22	*K. pneumoniae*	Raw milk	CFZ, CEF, FOX, CAZ, CTX, CRO, SXT, TGC, CIP, TPZ, AM, AMC, TET, FOF, CST	0.63	128	*mcr-2*	MW811416	*entB, mrkD, fimH, ybtS, irp1*
23	*K. pneumoniae*	Raw milk	CFZ, CEF, FOX, ATM, AMC, FOF, CST	0.29	4	*mcr-1*	MW811400	*entB, mrkD, ybtS, irp1*
24	*K. pneumoniae*	Raw milk	CFZ, CEF, FOX, CAZ, CTX, CRO, FEP, TPZ, AM, AMC, FOF, CST	0.5	128	*mcr-1*	MW811401	*entB, alls, mrkD, fimH, irp1*
25	*K. pneumoniae*	Clinical mastitis	CFZ, CEF, FOX, CAZ, CTX, CRO, FEP, SXT, TGC, TPZ, AM, AMC, FOF, CST	0.58	64	*mcr-3*	MW811428	*entB, alls, mrkD, fimH, irp1*
26	*K. pneumoniae*	Raw milk	CFZ, CEF, FOX, CAZ, CTX, CRO, FEP, TGC, TPZ, AM, AMC, FOF, CST	0.54	64	*mcr-1*	MW811402	*entB, mrkD, fimH, ybtS, irp1*
27	*K. pneumoniae*	Raw milk	CFZ, CEF, FOX, CAZ, CTX, CRO, TGC, TPZ, AM, AMC, TET, FOF, CST	0.54	8	*mcr-3*	MW811429	*entB, alls, mrkD, fimH, ybtS*
28	*K. pneumoniae*	Subclinical mastitis	CFZ, CEF, FOX, CAZ, CTX, CRO, TGC, TPZ, AM, AMC, FOF, CST	0.5	16	*mcr-1*	MW811403	*entB, fimH, ybtS, irp1*
29	*K. pneumoniae*	Subclinical mastitis	CFZ, CEF, FOX, CAZ, CTX, CRO, TOB, TGC, TPZ, AM, AMC, TET, FOF, CST	0.58	32	*mcr-2*	MW811417	*entB, mrkD, fimH, ybtS, irp1*
30	*P. aeuriginosa*	Clinical mastitis	CEF, FOX, CAZ, CFZ, NA, CIP, LVX, SXT, TGC, AM, AMC, CHL, FOF, CST, TET	0.63	>128	*mcr-7*	MW811434	*toxA*, *exoS*, *lasB*, *exoU*
**31**	*P. aeuriginosa*	Clinical mastitis	AK, CN, TOB, CEF, CFZ, FOX, CTX, CRO, NA, CIP, LVX, SXT, AM, AMC, CHL, FOF, TGC, CST, TET	0.79	128	*mcr-2*	MW811418	*toxA*, *exoS*, *lasB*, *exoU*, *phzM*
32	*P. aeuriginosa*	Subclinical mastitis	TOB, CEF, FOX, CAZ, CFZ, NA, AM, AMC, SXT, CHL, FOF, TET, CST	0.54	32	*mcr-1*	MW811404	*toxA*, *exoS*, *lasB*
33	*P. aeuriginosa*	Clinical mastitis	AK, CN, TOB, CEF, FOX, CAZ, CFZ, SXT, NA, CIP, LVX, AM, AMC, CHL, FOF, TET, CST	0.71	64	*mcr-3*	MW811430	*toxA*, *exoS*, *exoU*
34	*P. aeuriginosa*	Subclinical mastitis	CEF, FOX, CFZ, NA, CIP, LVX, SXT, AM, AMC, CHL, FOF, TET, CST	0.54	64	*mcr-3*	MW811431	*toxA*, *exoS*, *lasB*
35	*P. aeuriginosa*	Clinical mastitis	TOB, CEF, FOX, CFZ, SXT, AM, AMC, CHL, FOF, TET, CST	0.45	32	*mcr-1*	MW811405	*toxA,lasB*, *exoU*
**36**	*P. aeuriginosa*	Clinical mastitis	AK, CN, CEF, FOX, CFZ, CRO, FEP, NA, CIP, LVX, SXT, TGC, AM, AMC, CHL, FOF, TET, CST	0.75	16	*mcr-3*	MW811432	*toxA*, *exoS*, *lasB*, *exoU*, *phzM*
**37**	*P. aeuriginosa*	Subclinical mastitis	AK, CN, CEF, FOX, CFZ, CAZ, CTX, FEP, NA, CIP, LVX, SXT, TGC, AM, AMC, CHL, FOF, TET, CST	0.79	>128	*mcr-1*	MW811406	*toxA*, *exoS*, *lasB*, *exoU*
38	*A. hydrophila*	Raw milk	CAZ, CTX, CEF, CRO, FOX, NA, SXT, AM, AMC, CHL, CST, TET	0.5	128	*mcr-1*	MZ648218	*aer*
39	*A. hydrophila*	Raw milk	CFZ, CEF, FOX, CAZ, CTX, CRO, NA, SXT, ATM, AM, CST, TET	0.5	16	*mcr-1*	MZ648219	*act*
40	*A. hydrophila*	Raw milk	TOB, CEF, FOX, CAZ, CTX, CRO, TGC, AM, AMC, FOF, CHL, CST, TET	0.54	32	*mcr-2*	MZ648224	*aer, lip*
41	*A. hydrophila*	Raw milk	TOB, CEF, CFZ, FOX, CAZ, CTX, CRO, FEP, NA, SXT, AM, AMC, CHL, CST, TET	0.63	64	*mcr-1*	MZ648220	*aer, lip*
42	*A. hydrophila*	Raw milk	CEF, FOX, CAZ, CTX, CRO, NA, SXT, CST, TET	0.38	64	*mcr-3*	MZ648226	*lip*
43	*A. hydrophila*	Raw milk	CN, CEF, FOX, CAZ, CFZ, CTX, CRO, NA, SXT, ATM, CST, TET	0.5	128	*mcr-3*	MZ648227	*aer*
44	*A. hydrophila*	Raw milk	CN, TOB, CEF, FOX, CAZ, CTX, CRO, NA, SXT, ATM, TPZ, AMC, CHL, CST, TET	0.63	128	*mcr-1*	MZ648221	*hlyA, aer*
45	*A. hydrophila*	Raw milk	CEF, FOX, CAZ, CFZ, CTX, CRO, NA, SXT, ATM, AMC, CST, TET	0.5	64	*mcr-1*	MZ648222	*act*
46	*A. hydrophila*	Raw milk	CEF, FOX, CAZ, CTX, CRO, FEP, NA, SXT, TGC, CST, TET	0.46	32	*mcr-2*	MZ648225	*aer*
47	*A. hydrophila*	Raw milk	CEF, FOX, CAZ, CFZ, CTX, CRO, NA, SXT, TGC, ATM, CST, TET	0.5	>128	*mcr-1*	MZ648223	*aer, ast*

AM, ampicillin; AMC, amoxicillin-clavulanic acid; TPZ, piperacillin-tazobactam; CFZ, cefazolin; CEF, cephalothin; FOX, cefoxitin; CAZ, ceftazidime; CTX, cefotaxime; CRO, ceftriaxone; FEB, cefepime; IPM, imipenem; CN, gentamicin; TOB, tobramycin; AK, amikacin; NA, nalidixic acid; CIP, ciprofloxacin; LVX, levofloxacin; CST, colistin; TET, tetracycline; TGC, tigecycline; CHL, chloramphenicol; SXT, sulfamethoxazole-trimethoprim; ATM, aztreonam; FOF, fosfomycin. All isolates are multidrug resistant (MDR) except the bold ones are extensive drug resistant (XDR).

A Venn diagram (**Supplementary Figure S1**) was generated to determine the shared *mcr* variants between colistin-resistant isolates from clinical mastitis, subclinical mastitis, and raw milk. *E. coli mcr-4* and both *mcr-7* and *mcr-2* of *P. aeruginosa* were unique for clinical mastitis; whereas *K. pneumonia mcr-3* and *A. hydrophila mcr-1* to *mcr-3* variants were exclusive for raw milk. Only *E. coli mcr-3* and *K. pneumonia mcr-2* were common among all sources.

### Phylogeny of Colistin Resistance *mcr* Variants

**Figure 2:**
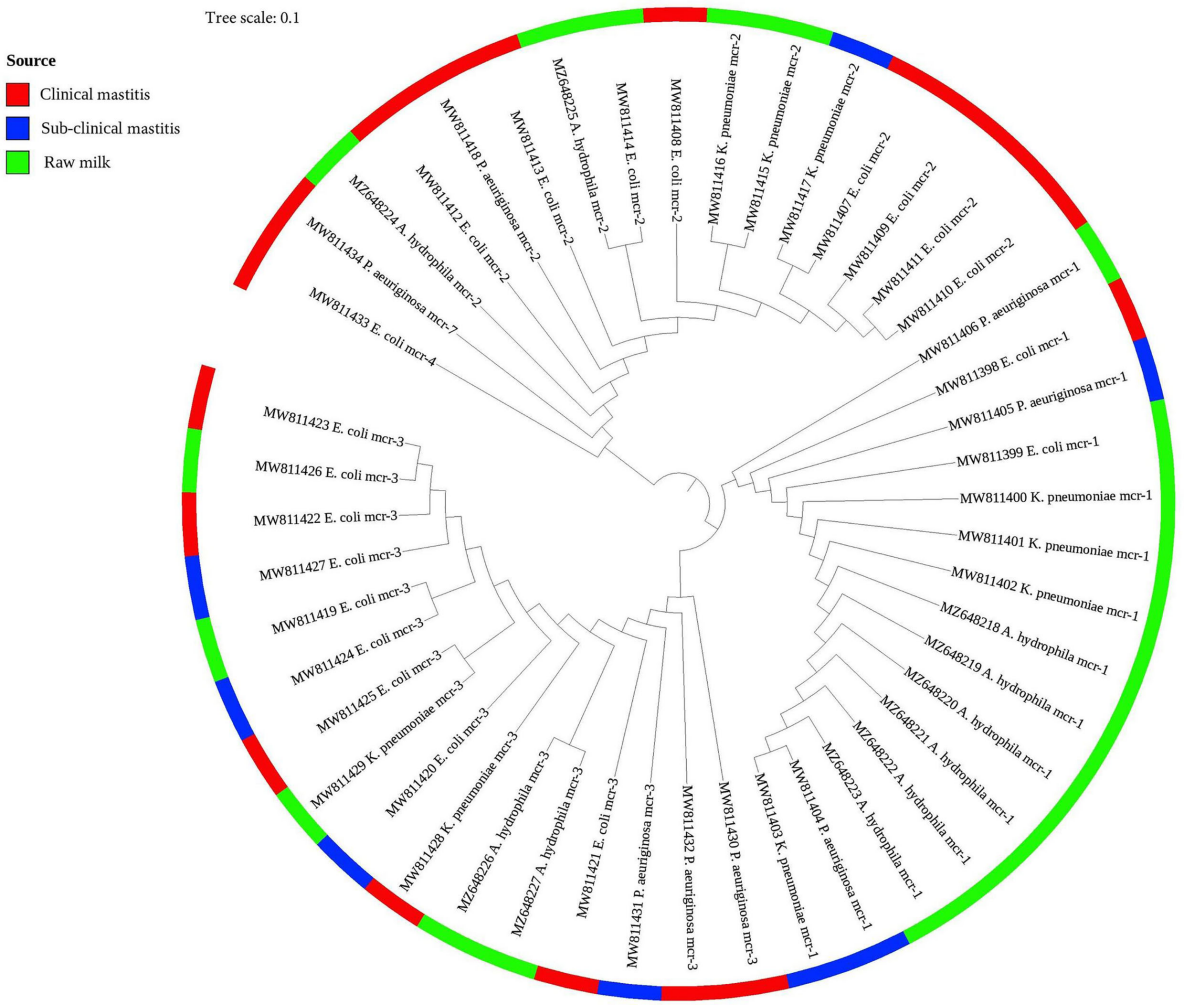
A phylogenetic tree shows the genetic relationship between *mcr* gene sequences of colistin-resistant Gram-negative bacteria recovered from clinical, subclinical mastitis, and raw milk. The tree was constructed using a neighbor-joining method with a bootstrap value for 1,000 replicates.

As presented in **Figure 2**, the phylogenetic tree was constructed based on nucleotide sequences for all identified colistin resistance *mcr* variants (*mcr-1* to *mcr-4* and *mcr-7*) in 47 Gram-negative bacteria isolates of various species. The phylogenetic analysis revealed five potential clusters indicating sequence heterogeneity among the *mcr* variants. Despite the overall diversity of bacterial isolates, which were originating from different on-farm locations/sources, the phylogenetic tree demonstrated the presence of the same clonal lineages of each *mcr* variant separately. Nonetheless, two *mcr* variants (*mcr-4*, accession number MW811433, and *mcr*-7, accession number MW811434) were clonally related; whereas the latter shared the same lineage with another *mcr-2* variant (accession number MW811412).

### Transferability of *mcr* Genes

**Figure 3:**
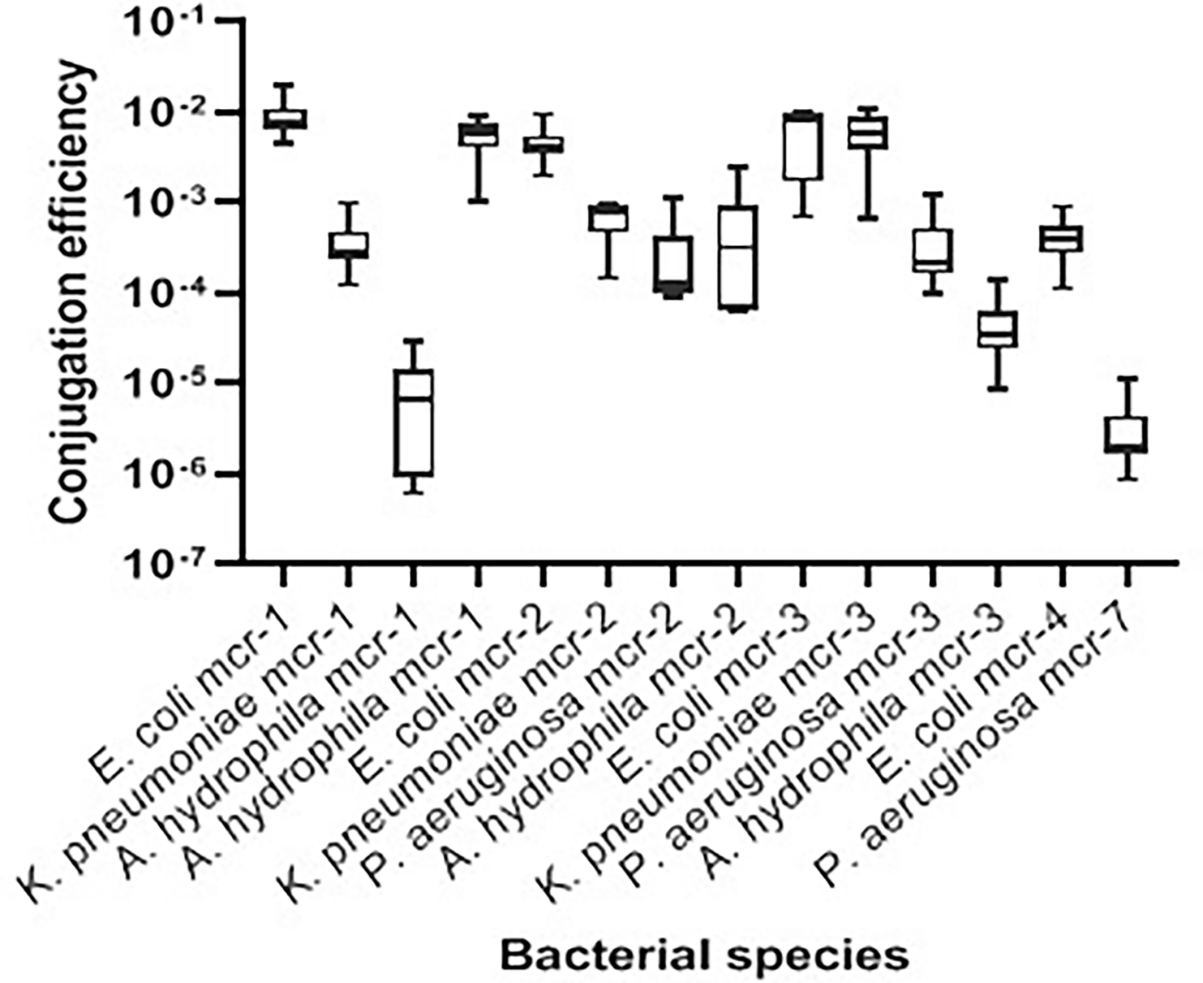
Transconjugation frequencies of 14 *mcr*-harboring Gram-negative isolates from bovine milk. The data are presented as mean values ± standard deviations. Code numbers of isolates are shown in **Table 2**.

Transconjugation assays revealed that representative Gram-negative isolates harboring *mcr-1*, *mcr-2*, *mcr-3*, *mcr-4*, and *mcr-7* were capable of transferring their genes to the *E. coli* J53-recipient strain. The conjugation efficiencies of these isolates ranged from 2.01 ± 0.816 × 10^−6^ to 9.50 ± 2.663 × 10^−3^ CFU/donor (**Table 3** and **Figure 3**). As expected, the recipient J53 strain did not grow on colistin and Na azide-incorporated media. The colistin MICs for transconjugants ranged from 4 to 64 µg/ml, demonstrating an increase of three to sevenfold relative to *E. coli* J53-recipient strain (MIC = 0.5 µg/ml) (**Table 3**).

**Table 3:** Conjugation efficiencies of some *mcr-1*-, *mcr-2*-, *mcr-3*-, *mcr-4*-, and *mcr-7*-positive isolates and colistin MIC values for transconjugants.

Bacterial species	Code No.	*mcr* gene	Transferability	Transconjugant MIC (µg/ml)^*^	CFU/ml			
					Recipient (*E. coli* J53)	Donor	Transconjugant	Conjugation efficiency
*E. coli*	9	*mcr-1*	+	16	2.62 × 10^8^	(3.14 ± 0.548) × 10^5^	(2.31 ± 0.142) × 10^3^	(7.36 ± 2.101) × 10^−3^
*K. pneumoniae*	26	*mcr-1*	+	32		(4.63 ± 0.856) × 10^6^	(1.36 ± 0.211) × 10^3^	(2.94 ± 0.621) × 10^−4^
*P. aeruginosa*	37	*mcr-1*	+	64		(7.12 ± 0.891) × 10^7^	(5.70 ± 1.224) × 10^2^	(8.01 ± 2.663) × 10^−6^
*A. hydrophila*	41	*mcr-1*	+	64		(5.53 ± 1.024) × 10^4^	(3.24 ± 0.882) × 10^2^	(5.86 ± 1.174) × 10^−3^
*E. coli*	10	*mcr-2*	+	16		(1.69 ± 0.442) × 10^5^	(7.01 ± 1.663) × 10^2^	(4.15 ± 1.111) × 10^−3^
*K. pneumoniae*	21	*mcr-2*	+	8		(5.46 ± 1.105) × 10^5^	(4.08 ± 1.022) × 10^2^	(7.47 ± 2.337) × 10^−4^
*P. aeruginosa*	31	*mcr-2*	+	32		(3.91 ± 0.462) × 10^6^	(4.11 ± 1.121) × 10^2^	(1.05 ± 0.184) × 10^−4^
*A. hydrophila*	40	*mcr-2*	+	16		(7.23 ± 1.335) × 10^5^	(2.64 ± 0.451) × 10^2^	(3.65 ± 0.326) × 10^−4^
*E. coli*	3	*mcr-3*	+	8		(3.61 ± 0.710) × 10^5^	(3.43 ± 0.352) × 10^3^	(9.50 ± 2.663) × 10^−3^
*K. pneumoniae*	27	*mcr-3*	+	4		(6.40 ± 0.633) × 10^6^	(4.26 ± 0.222) × 10^4^	(6.66 ± 1.812) × 10^−3^
*P. aeruginosa*	34	*mcr-3*	+	32		(3.12 ± 0.223) × 10^7^	(6.47 ± 1.362) × 10^3^	(2.07 ± 0.634) × 10^−4^
*A. hydrophila*	42	*mcr-3*	+	32		(6.46 ± 0.384) × 10^6^	(2.23 ± 0.331) × 10^2^	(3.45 ± 1.125) × 10^−5^
*E. coli*	7	*mcr-4*	+	16		(4.15 ± 0.361) × 10^6^	(1.81 ± 0.114) × 10^3^	(4.36 ± 1.632) × 10^−4^
*P. aeruginosa*	30	*mcr-7*	+	64		(5.46 ± 0.966) × 10^7^	(1.30 ± 0.314) × 10^2^	(2.01 ± 0.816) × 10^−6^

^*^Colistin MIC value = 0.5 µg/ml.

Conjugation efficiency = number of transconjugants/number of donor cells.

CFU, colony-forming unit.

CFU values were retrieved from duplicate measurements from two independent experiments.

### Virulence Characteristics of Colistin-Resistant Gram-Negative Bacteria

The profiles of virulence-associated genes detected in 47 colistin-resistant isolates are depicted in **Table 2**. Different combinations of virulence genes were observed among colistin-resistant isolates with almost all isolates harboring genes ranging from one to six. *LpfA* was the most frequently detected gene in the tested *E. coli* isolates (20/20; 100%) followed by *hlyF* (17/20; 85%), *iroN* (16/20; 80%), *iss* (15/20; 75%), *ireA* (13/20; 65%), and *traT* (12/20; 60%). The *entB* gene was detected in all nine colistin-resistant *K. pneumoniae* isolates examined; both *mrkD* and *fimH* were identified in 88.8%, *ybtS* and *irp1* in 77.7%, and *alls* in 44.4% of *K. pneumonia* isolates. The frequencies of *toxA*, *exoS*, *lasB*, *exoU*, and *phzM* genes in eight colistin-resistant *P. aeruginosa* isolates were 100%, 87.5%, 87.5%, 75%, and 25%, respectively. The *aer* gene is the most prevalent (70%) in *A. hydrophila* isolates followed by *lip* (30%) and *act* (20%). Both *a*s*t* and *hlyA* genes were detected in one *A. hydrophila* isolate (10%). There are significant differences in the frequencies of virulence genes among *E. coli* isolates (*p* = 0.04), *A. hydrophila* (*p* = 0.01), and *P. aeruginosa* (*p* = 0.005).

### Correlations Between the Antimicrobial Resistance and Virulence Traits

The correlation plots among pairs of antimicrobial resistance phenotypes, colistin resistance genes (*mcr*), and virulence genes in *E. coli*, *K. pneumoniae*, *P. aeruginosa*, and *A. hydrophila* are displayed in **Supplementary Figure S2** and **Supplementary Tables S2A–H**. Significant positive correlations were pronounced between fluoroquinolone resistance and the existence of *mcr-1* gene in *E. coli* (*r* = 0.58–0.67; *p* < 0.001). On the other hand, a strong positive correlation was significantly detected between a *fimH* virulence gene and the resistance to certain antimicrobials; ceftazidime, cefotaxime, ceftriaxone, and piperacillin-tazobactam in *K. pneumonia* isolates (*r* = 1; *p* < 0.001). Correlations between virulence traits and antimicrobial resistance were obvious in *P. aeruginosa* and *A. hydrophilus* isolates. Concerning *P. aeruginosa*, non-significant positive correlations were found between the analyzed virulence genes and antimicrobial agents/genes including the last-resort ones. The correlations between *exoU* and *phzM* virulence genes and gentamicin (*r* = 0.58; *p* = 0.1), *exoS* and fluoroquinolones (*r* = 0.65; *p* = 0.07), *exoU* and tigecycline (*r* = 0.58; *p* = 0.1), *phzM* and *mcr2* (*r* = 0.65; *p* = 0.07), and *phzM* and tigecycline (*r* = 0.58; *p* = 0.1) were mostly distinct. For *A. hydrophila*, the *hlyA* gene showed significant positive correlations with tobramycin (*r* = 0.67; *p* = 0.03) or piperacillin-tazobactam (*r* = 1; *p* < 0.001). Of interest, significant strong positive correlations were detected between the last resort antimicrobials; *mcr-2* and tigecycline (*r* = 0.76; *p* = 0.01) and *mcr-2* and fosfomycin (*r* = 0.67; *p* = 0.03).

### Efficacy of Disinfectants on Colistin-Resistant Gram-Negative Bacteria

According to the agar well-diffusion results, certain disinfectants had a wide efficacy against the test bacteria. However, some disinfectants were ineffective even at high concentrations (**Supplementary Table S3**). *E. coli* and *K. pneumonia* isolates were resistant to chlorohexdine gluconate at concentrations lower than 50% (MIC = 40%), whereas *P. aeruginosa* and *A. hydrophila* were sensitive to such disinfectant even at low concentrations (MIC = 10%). *E. coli* and *K. pneumoniae* were sensitive to iodine at high concentrations (MIC = 80%), while *P. aeruginosa* and *A. hydrophila* exhibited resistance at all tested concentrations. On the other hand, hydrogen peroxide had the best inhibitory effect against all tested bacterial isolates even at a low concentration (10%), except for *E. coli*, which was sensitive to H_2_O_2_ at a concentration of 50%. All tested bacteria were resistant to ethanol at all concentrations.

## Discussion

The recent global dissemination of *mcr* genes has raised the alarm of colistin resistance as a serious and urgent threat to public health (WHO, 2019). Consequently, large numbers of studies were carried out to explore the distribution of *mcr* genes in different reservoirs. Herein, we studied colistin-resistant Gram-negative bacteria isolated from bovine milk concerning the *mcr* resistance determinants and virulence characteristics. Unfortunately, 52% of the tested isolates were colistin resistant. The high proportion of colistin-resistant bacteria and the diversity of *mcr* variants detected here indicates a lot of independent acquisitions of *mcr* genes by colistin-sensitive bacteria and confirm the hypothesis that animals and food chain may play a key role in *mcr* transmission (Luo et al., 2020). This is considering an alarming ratio since previous studies have detected colistin resistance in 37%, 40%, and 41% of bacterial isolates from pigs, poultry, and human clinical samples, respectively (Savin et al., 2020; Lay et al., 2021; Qadi et al., 2021). Of note, we identified these isolates in milk samples, which included various bacterial species. These findings could be attributed to the frequent usage of colistin in dairy farms that in turn could be responsible for the development of colistin resistance. This is consistent with a previous report of a Canadian dairy farm, which uses colistin to increase the milk yield although its usage is not officially licensed in veterinary medicine (Saini et al., 2012; Webb et al., 2017).

Colistin-resistant *E. coli* isolates exhibited resistance to cephalothin, cefazolin, ceftazidime, ampicillin, amoxicillin-clavulanic acid, cefoxitin, cefotaxime, piperacillin-tazobactam, and fosfomycin. Concurrently, nearly similar patterns were obtained recently for *E. coli* against several antibiotics in India, Croatia, and Egypt (Zdolec et al., 2016; Singh et al., 2018; Ramadan et al., 2021; Soliman et al., 2021). For *K. pneumoniae* isolates, the antibiotic resistance pattern was closely like the pattern of Brazilian MDR *K. pneumoniae* isolates (Ferreira et al., 2019). They showed resistance to cefazolin, cephalothin, cefoxitin, ceftazidime, cefotaxime, ceftriaxone, sulfamethoxazole-trimethoprim, and tigecycline. On the contrary, our colistin-resistant *P. aeruginosa* isolates showed resistance to most groups of antibiotics; however, previous colistin-resistant *P. aeruginosa* isolates from Iran showed only resistance to ticarcillin, ciprofloxacin, aztreonam, ceftazidime, gentamicin, imipenem, and piperacillin/tazobactam (Goli et al., 2016). Our *A. hydrophila* isolates have an antimicrobial resistance pattern much akin to the antibiotic resistance profiles of pathogenic aeromonads isolated from ornamental fish with resistance to gentamicin, cephalothin, cefoxitin, ceftazidime, cefotaxime, ceftriaxone, nalidixic acid, sulfamethoxazole-trimethoprim, aztreonam, and chloramphenicol (Saengsitthisak et al., 2020). Colistin-resistant isolates found in this study exhibited extreme emergence and spread of antimicrobial resistance and high virulence as well.

It is almost established that *E. coli* is deemed to be the top-most bacteria for the *mcr* genes by carrying *mcr-1* up to *mcr-5* with the chance for coexistence of more than one *mcr* gene (Yin et al., 2017; Hammerl et al., 2018). Similarly, colistin-resistant *E. coli* isolates in our study were found to carry *mcr-1*, *mcr-2*, *mcr-3*, or *mcr-4* genes; 20% out of which were from raw milk and carried *mcr-1*, *mcr-2*, or *mcr-3* genes. It is the first detection of *mcr*-carrying isolates in raw milk samples. A previous study has identified *mcr-1* gene in one *E. coli* isolate from a cow suffering from subclinical mastitis in Egypt (Khalifa et al., 2016). Although the *mcr-1* gene was detected frequently from different samples (Khalifa et al., 2016; Lima Barbieri et al., 2017; Ramadan et al., 2021), it is foremost found here in the raw milk samples. Marvelously, one *E. coli* isolate carrying *mcr-1* gene obtained from hard cheese made from raw milk was reported in Egypt very lately (Ombarak et al., 2021), which in turn lightens the importance of raw milk quality control checks in Egypt, focusing on farm workers as well as the animal itself.

The prevalence of *mcr-3* gene in our isolated *E. coli* was the dominant one, followed by the *mcr-2* gene. Only one *E. coli* isolate was *mcr-4* positive. However, in this study, the *mcr-1*, *mcr-2*, and *mcr-3* genes were found in *E. coli* isolated from raw milk as well as clinical and subclinical mastitis samples; however, the only *mcr-4*-positive *E. coli* was isolated from a clinical mastitis sample. The variants *mcr-2*, *mcr-3*, and *mcr-4* were identified formerly in *E. coli* isolates from Belgium, China, and Italy, respectively, and were isolated from calves and pigs (Xavier et al., 2016; Carattoli et al., 2017; Yin et al., 2017). All these data confirmed that *E. coli* is the chief bacteria carrying *mcr* genes from different samples and in different geographical areas.

Notably, all our colistin-resistant *A. hydrophila* were isolated from raw milk samples, and the predominant gene in them was *mcr-1* followed by *mcr-2* and *mcr-3*. In contrast, colistin-resistant *A. hydrophila* were found to harbor *mcr-7* gene in cooked and raw meats and in environmental samples (Liu Y. et al., 2020). No previous reports until now showed the isolation of colistin-resistant *A. hydrophila* from raw milk samples. Our findings warrant that the source of raw milk contamination is most probably the farm workers rather than the animal source, particularly, that *mcr-3* gene was previously identified in isolates from human rectal swabs (Shen et al., 2018).

Of the identified colistin-resistant isolates, 19.12% were *K. pneumoniae* and were carrying *mcr-1*, *mcr-2*, or *mcr-3* genes. The three variants were identified in the isolates from raw milk samples, while *mcr-1* was found in subclinical mastitis isolates and *mcr-2* in both isolates from clinical and subclinical mastitis. It is almost agreed that the *mcr-1* gene is one of the few and clear examples of animal origin of a resistance trait that could hit the entire human health system (Poirel and Nordmann, 2016). Appealingly, three *mcr-1*-positive *K. pneumoniae* isolates were isolated from raw milk samples ensuring the role of humans in contamination and the transmission from animals to humans.

On the other hand, no colistin-resistant *P. aeruginosa* were found in the raw milk samples. They were isolated only from clinical and subclinical mastitis and were carrying *mcr-1*, *mcr-2*, *mcr-3*, or *mcr-7* genes. Although, we detected 37.5% and 12.5% of the colistin-resistant *P. aeruginosa* isolates carrying *mcr-1* and *mcr-2* genes; respectively, it was found lately in Egypt that colistin-resistant *P. aeruginosa* were negative for *mcr-2* gene and 50% were positive for *mcr-1* (Abd El-Baky et al., 2020). We also found that the *mcr-3* variant was prevalent with 37.5% among our colistin-resistant *P. aeruginosa* isolates. Even, to our knowledge, there is no report detected on *mcr-3* in *P. aeruginosa* isolates before. Moreover, this is the first-time reporting *mcr-7* from Egyptian isolates, and it was identified in *P. aeuriginosa* from clinical mastitic milk. The first plasmid-mediated colistin resistance gene *mcr-7* was detected in *K. pneumoniae* from chickens in China (Yang et al., 2018). Recently, the *mcr-7* gene was detected in a water sample from an environment of an alligator (dos Santos et al., 2020). The detection of *mcr-7* in our isolate from clinical mastitis confirms the dissemination of *mcr* gene and its variants at all levels; humans, animals, and environmental. This was confirmed here while application of the conjugation assay as the plasmid harboring *mcr* genes (*mcr-1* to *mcr-4*, and *mcr*-7) successfully transferred colistin resistance to the recipient strain through a broth-mating assay, representing their capability to mobilize the *mcr*-genes between isolates (Migura-Garcia et al., 2020).

The virulome of our colistin-resistant isolates was screened in each species. Many of the identified virulence genes in our study were required for bacterial survival and had a role in causing mastitis (Tartor and El-Naenaeey, 2016; Gao et al., 2019; Guerra et al., 2020). This in turn raises the alarm that most of the colistin-resistant Gram-negative isolates are highly pathogenic. Here, the establishment of an interplay between antimicrobial resistance and virulence traits corresponded to each analyzed Gram-negative bacteria, revealing positive correlations between the existence of certain virulence genes and resistance to antimicrobial agents/genes. A previous study suggested that the mobile genetic elements of proficient pathogens favor the coselection of both resistance and virulence-associated traits (Hennequin and Robin, 2016).

The virulome, as well as the resistome patterns of our colistin-resistant isolates, confirmed the elevated pathogenicity and the widespread antibiotic resistance that correlated to these isolates. The phylogenetic analysis of the nucleotide sequences of our colistin resistance *mcr* variants showed three distinguishable clusters. Interestingly, each cluster has the same *mcr* variant, and this is in accordance with what was reported before about *mcr-1*, *mcr-2*, and *mcr-3* (Zhang et al., 2018). The presence of each variant of the *mcr* gene in the same clonal lineage despite the different sources of isolation confirms the high spread of colistin resistance from one source to another. Each of the *mcr-4* and *mcr-7* variants was shown in a unique cluster. This was unsurprising since our study showed the first report of *mcr-7* in *P. aeruginosa*. Similarly, *mcr-4* was previously found in a unique phylogenetic group (Zhang et al., 2019). Our findings proved that each *mcr* gene has a distinct evolution and spread path producing a characteristic phenotypic colistin resistance.

All the above information has raised the alert for controlling the spread of colistin-resistant isolates. Thus, we decided to evaluate four commonly used biocides in the dairy farms against colistin-resistant isolates. In accordance with Lanjri et al. (2017), all isolates were sensitive to chlorhexidine with different concentrations. This is attributed to its cationic nature that helps in electrostatic interaction with the anionic group lipopolysaccharide in the cytoplasmic membrane of Gram-negative bacteria, resulting in modification of the membrane permeability and causing membrane disruption and cell death due to coagulation of the cytoplasm (Estrela et al., 2003). Another tested biocide was iodine; it showed an inhibitory effect on *E. coli* and *K. pneumoniae* at high concentrations and no inhibition effect on *P. aeuriginosa* and *A. hydrophila* at all tested concentrations. Iodine is proven to be considered a broad-spectrum, rapidly acting bactericidal, and effective against all mastitis-causing bacteria (Gleeson et al., 2009). It acts through its penetration into the proteins, nucleotides, and fatty acids of microorganisms causing cell death (Gupta et al., 2018), which makes the development of resistance unlikely. However, in the field, the presence of some residues in the environment like the organic matter of the teat skin or in the challenge suspension may have been reacting with the disinfectant and thus less germicidal activity may have been produced (Enger et al., 2015). These conditions permit some bacteria to be exposed too much to lower concentrations of the active ingredients consequently and thus increasing their resistance to the used disinfectants (Wales and Davies, 2015). These results were in agreement with what was reported earlier that a dip containing the highest free iodine provided a greater significantly superior reduction of pathogens compared with a lower concentration of iodine dip (Foret et al., 2005). Furthermore, all isolates showed a sensitivity to H_2_O_2_, and this had been confirmed previously. H_2_O_2_ produces oxidants as singlet oxygen, superoxide radicals, and hydroxyl radicals with high penetration power and not specific in the points of contact by attacking bacteria at any essential cell components, leading to a decrease in the probability of bacteria to create resistance with these compounds (Nilima, 2011; Benavides, 2014). This was in contrast with Enger et al. (2015) who reported that H_2_O_2_ was the least efficacious tested teat dip, and these differences may be due to the evolution of resistance by the gradual use of the disinfectant (Wales and Davies, 2015). However, different concentrations of ethanol showed no antimicrobial activity against all tested bacteria, and this is in agreement with what was proved before about ethanol effectiveness (Al-Dabbbagh et al., 2015). This ethanol tolerance mainly appears to result in large part from adaptive and evolutionary changes in cell membrane composition (Ingram, 1990).

## Conclusion

Ultimately, the spread of mobile *mcr* genes between MDR and XDR virulent Gram-negative isolates from dairy cattle confirms the dissemination of the *mcr* gene and its variants at all levels: human, animal, and environmental. Consequently, a decision to stop using colistin in food animals is a principal demand to defy XDR and MDR emergence. Additionally, establishing a perfect quality control check system in Egypt, focusing on both farm workers and the animal itself is urgent.

## Data Availability Statement

The datasets presented in this study can be found in online repositories. The names of the repository/repositories and accession number(s) can be found in the article/**Supplementary Material**.

## Author Contributions

YT and NA contributed equally in the conception and design of the study and participated with RG, HE, EK, and AA in the application of classical microbiological techniques. YT and NA carried out the PCR assays, sequencing approaches, and participated with HR in sequence analysis. HR performed the bioinformatics and statistical analyses of the data. RG, HE, SE, EK, AA, SA, and HR conceived the study and participated in the design. YT, RG, NA, HE, SE, and AA wrote the initial draft of the manuscript. All authors revised the manuscript critically for important intellectual content and gave the final approval of the version to be published.

## Conflict of Interest

The authors declare that the research was conducted in the absence of any commercial or financial relationships that could be construed as a potential conflict of interest.

## Publisher’s Note

All claims expressed in this article are solely those of the authors and do not necessarily represent those of their affiliated organizations, or those of the publisher, the editors and the reviewers. Any product that may be evaluated in this article, or claim that may be made by its manufacturer, is not guaranteed or endorsed by the publisher.

## Supplementary Material

Supplementary Figure S1
**|** A Venn diagram showing the intersection between the *mcr* variants of colistin-resistant isolates from clinical, subclinical mastitis, and raw milk.

Supplementary Figure S2
**|** Correlation plot between antimicrobial resistance phenotypes, colistin resistance genes (*mcr*) and virulence genes in *E. coli*, *K. pneumoniae*, *P. aeruginosa*, and *A. hydrophila*. In each plot, each cell represents the *R*-value (correlation coefficient) for each pair of features. The color and circle size indicate the value of correlation (red = negative and blue = positive correlation). The more intense the color, the higher the correlation value. Stars referring to significant correlation determined at a cutoff = 0.05. Insignificant correlations are left without stars.
