# Supplementary figures and images for: Virulence Determinants and Plasmid-Mediated Colistin Resistance mcr Genes in Gram-Negative Bacteria Isolated From Bovine Milk
Source: Front Cell Infect Microbiol. 2021 Nov 23;11:761417. doi: 10.3389/fcimb.2021.761417 (PMC8650641; doi:10.3389/fcimb.2021.761417)

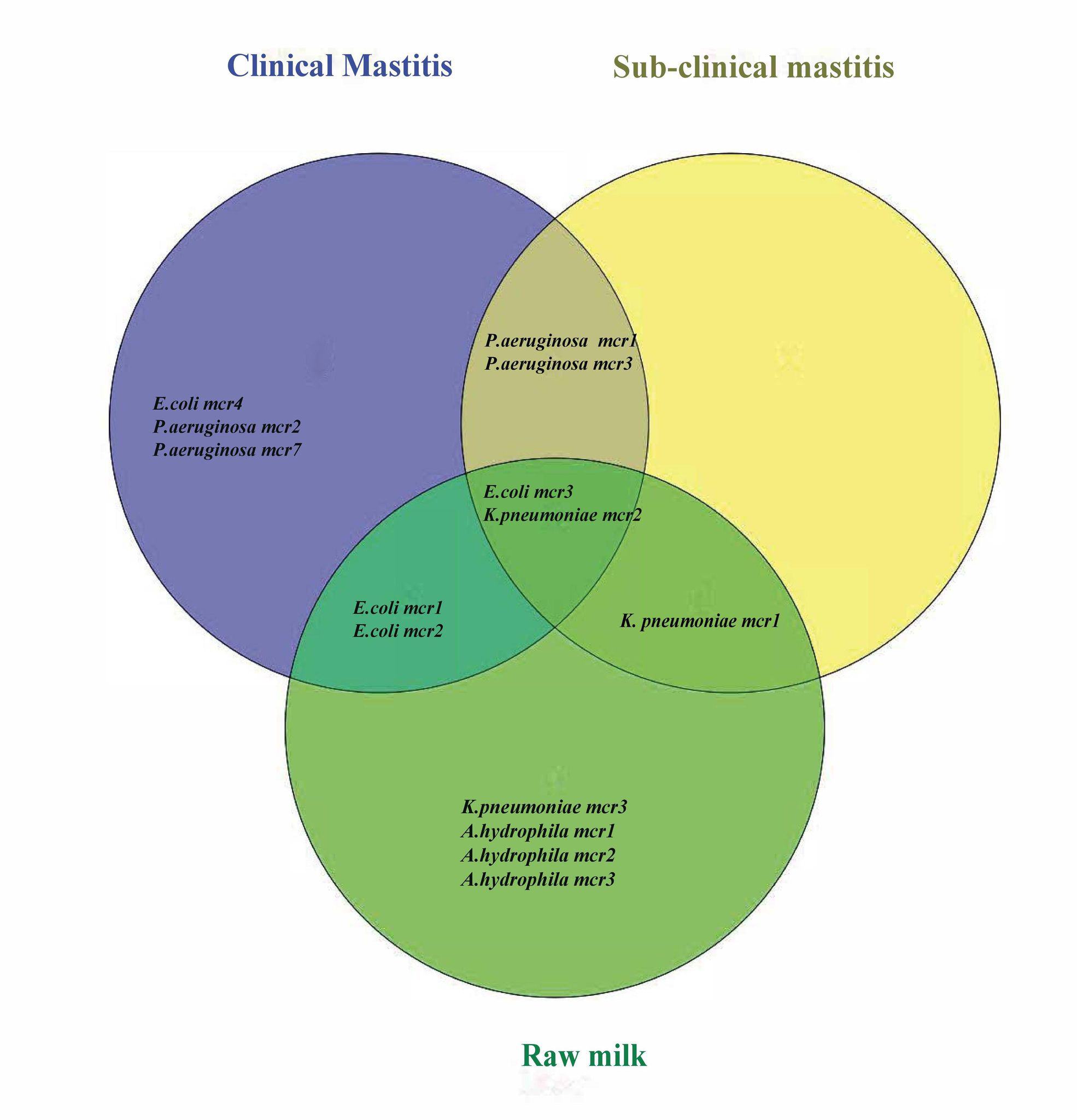

Supplement: Supplementary Figure S1 — | A Venn diagram showing the intersection between the mcr variants of colistin-resistant isolates from clinical, subclinical mastitis, and raw milk. [file Image_1.jpeg]

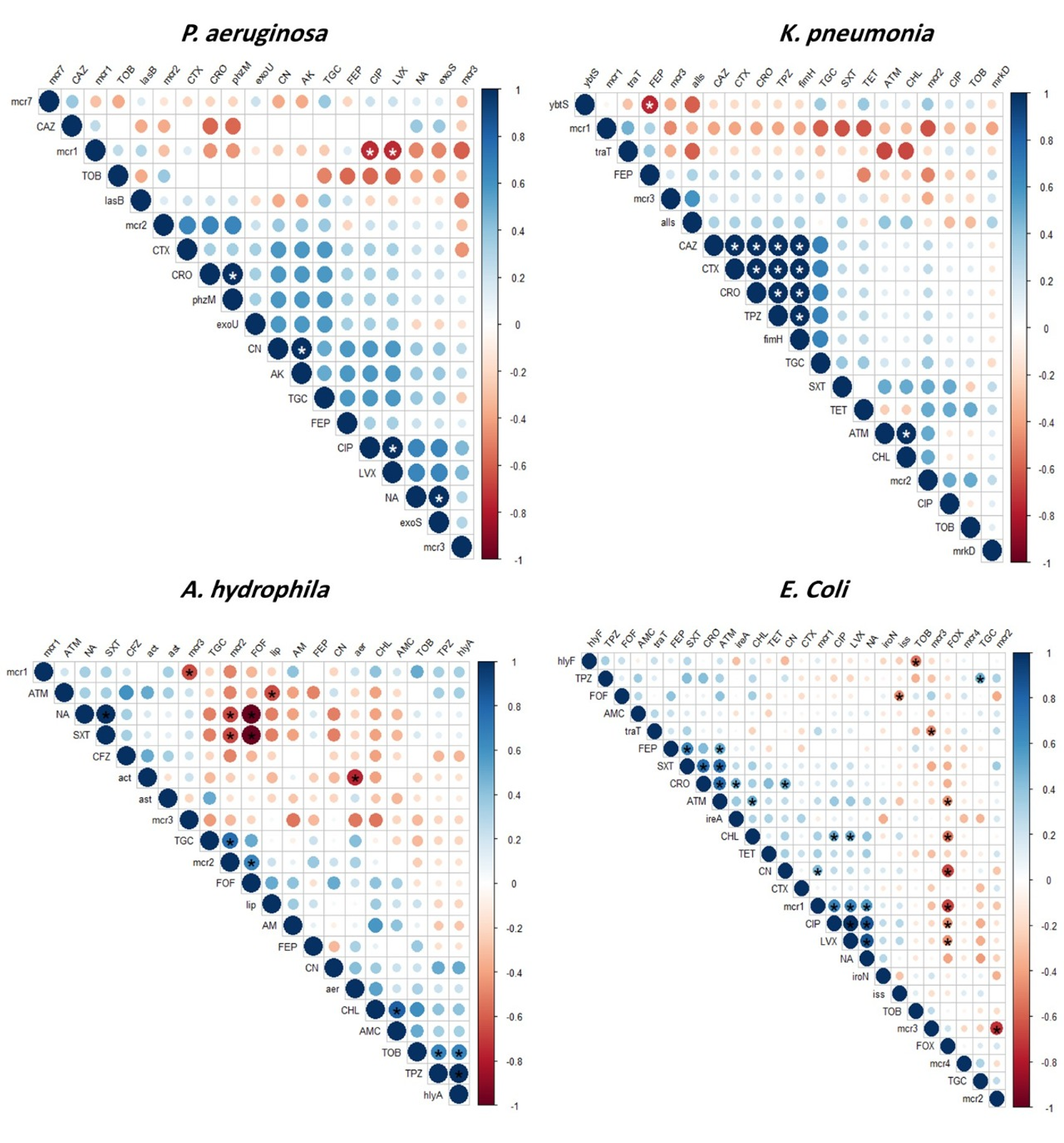

Supplement: Supplementary Figure S2 — | Correlation plot between antimicrobial resistance phenotypes, colistin resistance genes (mcr) and virulence genes in E. coli, K. pneumoniae, P. aeruginosa, and A. hydrophila. In each plot, each cell represents the R-value (correlation coefficient) for each pair of features. The color and circle size indicate the value of correlation (red = negative and blue = positive correlation). The more intense the color, the higher the correlation value. Stars referring to significant correlation determined at a cutoff = 0.05. Insignificant correlations are left without stars. [file Image_2.png]
