# Supplementary material for: Virulence Determinants and Plasmid-Mediated Colistin Resistance mcr Genes in Gram-Negative Bacteria Isolated From Bovine Milk
Source: Front Cell Infect Microbiol. 2021 Nov 23;11:761417. doi: 10.3389/fcimb.2021.761417 (PMC8650641; doi:10.3389/fcimb.2021.761417)
Supplement: Supplementary file 3 [file Table_1.doc]

**Table S1: Oligonucleotide primer sequences used in this study**

| **Primer use and target gene** | **Nucleotide sequence (5′→3′)** | **Amplicon size (bp)** | **Annealing temperature (^◦^C)** | **Reference** |
| --- | --- | --- | --- | --- |
| **Bacterial identification:** |  |  |  |  |
| *E. coli uidA* | F: TATGGAATTTCGCCGATTTT  R: TGTTTGCCTCCCTGCTGCGG | 166 | 55 | [Heijnen](https://pubmed.ncbi.nlm.nih.gov/?term=Heijnen+L&cauthor_id=17176819)  and  [Medema](https://pubmed.ncbi.nlm.nih.gov/?term=Medema+G&cauthor_id=17176819) (2006) |
| *K. pneumoniae 16S-23S ITS* | F: ATTTGAAGAGGTTGCAAACGAT  R: TTCACTCTGAAGTTTTCTTGTGTTC | 130 | 55 | Liu et al. (2008) |
| *Citrobacter* species*16S rRNA* | F: GCTCAACCTGGGAACTGCATCCGA  R: AGTTCCGGCCTAACCGCTGGCAA | 529 | 58 | Anbazhagan et al. (2010) |
| *Enterobacter cloacae atpd* | F:CGAGAGCCTGGTGCTGC  R: GATTGGCTGACCCAAT | 180 | 58 | Anbazhagan et al. (2010) |
| *Pseudomonas aeruginosa oprL* | F: ATGGAAATGCTGAAATTCGGC  R: CTTCTTCAGCTCGACGCGACG | 504 | 55 | Xu et al. (2004) |
| *Aeromonas hydrophila* 16S rRNA | F: GAAAGGTTGATGCCTAATACGTA  R: CGTGCTGGCAACAAAGGACAG | 625 | 50 | Gordon et al. (2007) |
| **Virulence genes**: |  |  |  |  |
| *- E. coli* |  |  |  |  |
| *hlyF* (Putative avian hemolysin) | F: GGCGATTTAGGCATTCCGATACTC  R: ACGGGGTCGCTAGTTAAGGAG | 599 | 60 | Johnson et al. (2006) |
| *IreA* (Iron-responsive element) | F: ATTGCCGTGATGTGTTCTGC  R: CACGGATCACTTCAATGCGT | 384 | 58 | Ewers et al. (2007) |
| *iroN* (Iron acquisition gene) | F: ATCCTCTGGTCGCTAACTG  R: CTGCACTGGAAGAACTGTTCT | 847 | 58 | Ewers et al. (2007) |
| *Iss* (Increased serum survival) | F: ATCACATAGGATTCTGCCG  R: CAGCGGAGTATAGATGCCA | 309 | 58 | Ewers et al. (2005) |
| *lpfA* (Long polar fimbriae) | F: ATGAAGCGTAATATTATAG  R: TTATTTCTTATATTCGAC | 573 | 52 | Doughty et al. (2002) |
| *traT* (Complement resistance protein precursor) | F: GTGGTGCGATGAGCACAG  R:TAGTTCACATCTTCCACCATCG | 430 | 58 | Ewers et al. (2007) |
| *- K. pneumoniae* |  |  |  |  |
| *entB* (Enterobactin biosynthesis gene) | F: CTGCTGGGAAAAGCGATTGTC  R: AAGGCGACTCAGGAGTGGCTT | 385 | 49 | Wasfi et al. (2016) |
| *alls* (Allantoin metabolism gene) | F: CATTACGCACCTTTGTCAGC  R: GAATGTGTCGGCGATCAGCTT | 764 | 60 | Compain et al. (2014) |
| *mrkD* (Type 3 fimbrial adhesin) | F: CCACCAACTATTCCCTCGAA  R: ATGGAACCCACATCGACATT | 226 | 43 | El Fertas-Aissani et al*.* (2013) |
| *fimH* (Fimbrial adhesin) | F: GCCAACGTCTACGTTAACCTG  R: ATATTTCACGGTGCCTGAAAA | 180 | 43 | Wasfi et al. (2016) |
| *ybtS* (Yersiniabactin) | F: GACGGAAACAGCACGGTAAA  R: GAGCATAATAAGGCGAAAGA | 242 | 60 | Compain et al. (2014) |
| *Irp-1* (Yersinibactin biosynthesis gene) | F: TGAATCGCGGGTGTCTTATGC  R: TCCCTCAATAAAGCCCACGCT | 238 | 49 | El Fertas-Aissani et al. (2013) |
| *- P. aeruginosa* enterotoxin genes |  |  |  |  |
| *exoU* | F: CCGTTGTGGTGCCGTTGAAG  R: CCAGATGTTCACCGACTCGC | 134 | 55 | Winstanley et al. (2005) |
| *exoS* | F: GCGAGGTCAGCAGAGTATCG  R: TTCGGCGTCACTGTGGATGC | 118 | 55 | Winstanley et al. (2005) |
| *lasB* | F: ACAGGTAGAACGCACGGTTG  R: GATCGACGTGTCCAAACTCC | 1220 | 54 | Finnan et al. (2004) |
| *toxA* | F: GACAACGCCCTCAGCATCACCAGC  R: CGCTGGCCCATTCGCTCCAGCGCT | 396 | 55 | Matar et al. (2002) |
| *phzM* | F: ATGGAGAGCGGGATCGACAG  R: ATGCGGGTTTCCATCGGCAG | 875 | 54 | Finnan et al. (2004) |
| *- A. hydrophila* |  |  |  |  |
| *hlyA* (Hemolysin) | F: GGCCGGTGGCCCGAAGATACGGG  R: GGCGGCGCCGGACGAGACGGGG | 592 | 62 | Zhu et al. (2007) |
| *Aer* (Aerolysin) | F: CCTATGGCCTGAGCGAGAAG  R: CCAGTTCCAGTCCCACCACT | 431 | 55.5 | Oliveira et al. (2012) |
| *Lip* (Lipase) | F: AACCTGGTTCCGCTCAAGCCGTTG  R: TTGCTCGCCTCGGCCCAGCAGCT | 760 | 62 | Cascon et al. (1996) |
| *Ast* (Cytotonic heat–stable enterotoxin) | F: TCTCCATGCTTCCCTTCCACT  R: GTGTAGGGATTGAAGAAGCCG | 331 | 55 | Sen and Rodgers (2004) |
| *Act*  (Cytotoxic enterotoxin) | F: GAGAAGGTGACCACCAAGAACA  R: AACTGACATCGGCCTTGAACTC | 232 | 42 | Hu et al. (2012) |
| **Detection of colistin resistance genes:** |  |  |  |  |
| *mcr-1* | F: CGGTCAGTCCGTTTGTTC  R: CTTGGTCGGTCTGTAGGG | 309 | 55 | Liu et al. (2016) |
| *mcr-2* | F: TGTTGCTTGTGCCGATTGGA  R: AGATGGTATTGTTGGTTGCTG | 567 | 65 | Xavier et al. (2016) |
| *mcr-3* | F: TTGGCACTGTATTTTGCATTT  R:TTAACGAAATTGGCTGGAACA | 542 | 50 | Yin et al. (2017) |
| *mcr-4* | F: ATTGGGATAGTCGCCTTTTT  R:TTACAGCCAGAATCATTATCA | 488 | 58 | Carattoli et al. (2017) |
| *mcr-5* | F: TATCTCGACAAGGCCATGCTG  R: GAATCTGGCGTTCGTCGTAGT | 613 | 50 | Borowiak et al. (2017) |
| *mcr-6* | F:GTCCGGTCAATCCCTATCTGT  R: ATCACGGGATTGACATAGCTAC | 556 | 55 | Yang et al. (2019) |
| *mcr-7.1* | F: AGGGGATAAACCGACCCTGA  R: TGATCTCGATGTTGGGCACC | 335 | 55 | Yang et al. (2018) |
| *mcr-8* | F: AACCGCCAGAGCACAGAATT  R: TTCCCCCAGCGATTCTCCAT | 667 | 60 | Yang et al. (2019) |
| *mcr-9* | F: GGTAGTTATTCCGCTGG  R: TCGCGGTCAGGATTAAC | 295 | 58 | Kieffer et al. (2019) |

F, forward; R, reverse; bp, base pair

**References**

Anbazhagan D, Kathirvalu G G, Mansor M, Yan G O S, Y. M. Y. and S. S. D. (2010). Multiplex Polymerase Chain Reaction (PCR) Assays for the Detection of *Enterobacteriaceae* in Clinical Samples. *African J. Microbiol. Res.* 4, 1186–1191.

Borowiak, M., Hammerl, J. A., Deneke, C., Fischer, J., Szabo, I., and Malorny, B. (2019). Characterization of *Mcr-5*-Harboring *Salmonella* *Enterica* Subsp. *Enterica* Serovar Typhimurium Isolates from Animal and Food Origin in Germany. *Antimicrob. Agents Chemother.* 63 (6), e00063-19. doi:10.1128/AAC.00063-19.

Carattoli, A., Villa, L., Feudi, C., Curcio, L., Orsini, S., Luppi, A., et al. (2017). Novel Plasmid-Mediated Colistin Resistance *Mcr*-*4* Gene in *Salmonella* and *Escherichia coli*, Italy 2013, Spain and Belgium, 2015 to 2016. *Eurosurveillance* 22 (31), 30589. doi:10.2807/1560-7917.ES.2017.22.31.30589.

Cascón, A., Anguita, J., Hernanz, C., Sánchez, M., Fernández, M., and Naharro, G. (1996). Identification of *Aeromonas hydrophila* Hybridization Group 1 by PCR Assays *Appl. Environ. Microbiol.* 62 (4), 1167–1170. doi:10.1128/AEM.62.4.1167-1170.1996.

Compain, F., Babosan, A., Brisse, S., Genel, N., Audo, J., Ailloud, F., et al. (2014). Multiplex PCR for Detection of Seven Virulence Factors and K1/K2 Capsular Serotypes of *Klebsiella pneumoniae*. *J. Clin. Microbiol.* 52 (12), 4377- 4380. doi:10.1128/JCM.02316-14.

Daling, Z., Aihua, L., Jianguo, W., Ming, L., Taozhen, C., and Jing, H. (2007). Correlation between the Distribution Pattern of Virulence Genes and Virulence of *Aeromonas hydrophila* Strains. *Front. Biol. China* 2 (2), 176–179. doi:10.1007/s11515-007-0024-4.

Doughty, S., Sloan, J., Bennett-Wood, V., Robertson, M., Robins-Browne, R. M., and Hartland, E. L. (2002). Identification of a Novel Fimbrial Gene Cluster Related to Long Polar Fimbriae in Locus of Enterocyte Effacement-Negative Strains of Enterohemorrhagic *Escherichia coli*. *Infect. Immun.* 70 (12), 6761–6769. doi:10.1128/IAI.70.12.6761-6769.2002.

El Fertas-Aissani, R., Messai, Y., Alouache, S., and Bakour, R. (2013). Virulence Profiles and Antibiotic Susceptibility Patterns of *Klebsiella pneumoniae* Strains Isolated from Different Clinical Specimens. *Pathol. Biol.* 61(5), 209–216. doi:10.1016/J.PATBIO.2012.10.004.

Ewers, C., Janssen, T., Kiessling, S., Philipp, H., and Wieler, L. (2005). Rapid Detection of Virulence-Associated Genes in Avian Pathogenic *Escherichia coli* by Multiplex Polymerase Chain Reaction. *Avian Dis.* 49 (2), 269–273. doi:10.1637/7293-102604R.

Ewers, C., Li, G., Wilking, H., Kießling, S., Alt, K., Antáo, E. M., et al. (2007). Avian Pathogenic, Uropathogenic, and Newborn Meningitis-Causing *Escherichia Coli*: How Closely Related Are They? *Int. J. Med. Microbiol.* 297 (3), 163–176. doi:10.1016/J.IJMM.2007.01.003.

Finnan, S., Morrissey, J. P., Gara, F. O., and Boyd, E. F. (2004). Genome Diversity of *Pseudomonas aeruginosa* Isolates from Cystic Fibrosis Patients and the Hospital Environment. 42 (12), 5783–5792. doi:10.1128/JCM.42.12.5783.

Gordon, L., Giraud, E., Ganière, J. P., Armand, F., Bouju-Albert, A., De La Cotte, N., et al. (2007). Antimicrobial Resistance Survey in a River Receiving Effluents from Freshwater Fish Farms. *J. Appl. Microbiol.* 102 (4), 1167–1176. doi:10.1111/j.1365-2672.2006.03138.x.

Heijnen L. and Medema G. (2006). Quantitative Detection of E. Coli, E. Coli O157 and Other Shiga Toxin Producing E. Coli in Water Samples Using a Culture Method Combined with Real-Time PCR. *J Water Heal.* 4, 487–498.

Hu, M., Wang, N., Pan, Z. H., Lu, C. P., and Liu, Y. J. (2012). Identity and Virulence Properties of *Aeromonas* Isolates from Diseased Fish, Healthy Controls and Water Environment in China. *Lett. Appl. Microbiol.* 55 (3), 224–233. doi:10.1111/J.1472-765X.2012.03281.X.

Johnson, T. J., Siek, K. E., Johnson, S. J., and Nolan, L. K. (2006). DNA Sequence of a ColV Plasmid and Prevalence of Selected Plasmid-Encoded Virulence Genes among Avian *Escherichia coli* Strains. *J. Bacteriol.* 188 (2), 745–758. doi:10.1128/JB.188.2.745-758.2006.

Kieffer, N., Royer, G., Decousser, J. W., Bourrel, A. S., Palmieri, M., De La Rosa, J. M. O., et al. (2019). *Mcr-9*, an Inducible Gene Encoding an Acquired Phosphoethanolamine Transferase in *Escherichia coli*, and Its Origin. *Antimicrob. Agents Chemother.* 63 (9), e00965-19. doi:10.1128/AAC.00965-19.

Liu, Y., Liu, C., Zheng, W., Zhang, X., Yu, J., Gao, Q., et al. (2008). PCR Detection of *Klebsiella pneumoniae* in Infant Formula Based on 16S-23S Internal Transcribed Spacer. *Int. J. Food Microbiol.* 125 (3), 230–235. doi:10.1016/j.ijfoodmicro.2008.03.005.

Liu, Y. Y., Wang, Y., Walsh, T. R., Yi, L. X., Zhang, R., Spencer, J., et al. (2016). Emergence of Plasmid-Mediated Colistin Resistance Mechanism *MCR-1* in Animals and Human Beings in China: A Microbiological and Molecular Biological Study. *Lancet Infect. Dis.* 16 (2), 161–168. doi:10.1016/S1473-3099(15)00424-7.

Matar, G. M., Ramlawi, F., Hijazi, N., Khneisser, I., and Abdelnoor, A. M. (2002). Transcription Levels of *Pseudomonas aeruginosa* Exotoxin A Gene and Severity of Symptoms in Patients with Otitis Externa. 45, 350–354. doi:10.1007/s00284-002-3703-z.

Oliveira, S. T. L., Veneroni-Gouveia, G., and Costa, M. M. (2012). Molecular Characterization of Virulence Factors in *Aeromonas* *hydrophila* Obtained from Fish. *Pesqui. Vet. Bras.* 32 (8), 701–706. doi:10.1590/S0100-736X2012000800004.

Sen, K., and Rodgers, M. (2004). Distribution of six virulence factors in *Aeromonas* species isolated from US drinking water utilities: a PCR identification. *J. Appl. Microbiol.* 97 (5), 1077–1086. doi:10.1111/J.1365-2672.2004.02398.X.

Wasfi, R., Elkhatib, W. F., and Ashour, H. M. (2016). Molecular Typing and Virulence Analysis of Multidrug Resistant *Klebsiella pneumoniae* Clinical Isolates Recovered from Egyptian Hospitals. *Sci. Rep.* 6, 38929. doi:10.1038/SREP38929.

Winstanley, C., Kaye, S. B., Neal, T. J., Chilton, H. J., Miksch, S., Hart, C. A., et al. (2005). Genotypic and Phenotypic Characteristics of *Pseudomonas aeruginosa* Isolates Associated with Ulcerative Keratitis. *J. Med. Microbiol.* 54 (6), 519–526. doi:10.1099/JMM.0.46005-0.

Xavier, B. B., Lammens, C., Ruhal, R., Malhotra-Kumar, S., Butaye, P., Goossens, H., et al. (2016). Identification of a Novel Plasmid-Mediated Colistin resistance Gene, *Mcr-2*, in *Escherichia coli*, Belgium, June 2016. *Euro surveill.*  21(27). doi:10.2807/1560-7917.ES.2016.21.27.30280.

Xu, J., Moore, J. E., Murphy, P. G., Millar, B. C., and Elborn, J. S. (2004). arly Detection of *Pseudomonas aeruginosa* - Comparison of Conventional versus Molecular (PCR) Detection Directly from Adult Patients with Cystic Fibrosis (CF). *Ann. Clin. Microbiol. Antimicrob.* 3, 21. doi:10.1186/1476-0711-3-21.

Yang, F., Shen, C., Zheng, X., Liu, Y., Ahmed, M. A. E. G. E. S., Zhao, Z., et al. (2019). Plasmid-Mediated Colistin Resistance Gene *Mcr-1* in *Escherichia Coli* and *Klebsiella pneumoniae* Isolated from Market Retail Fruits in Guangzhou, China. *Infect. Drug Resist.* 12, 385–389. doi:10.2147/IDR.S194635.

Yang, Y. Q., Li, Y. X., Lei, C. W., Zhang, A. Y., and Wang, H. N. (2018). Novel Plasmid-Mediated Colistin Resistance Gene *Mcr-7.1* in *Klebsiella pneumoniae*. *J. Antimicrob. Chemother.* 73 (7), 1791–1795. doi:10.1093/jac/dky111.

Yin, W., Li, H., Shen, Y., Liu, Z., Wang, S., Shen, Z., et al. (2017). Novel Plasmid-Mediated Colistin Resistance Gene *Mcr-3* in Escherichia coli. *MBio* 8 (3), 543–560. doi:10.1128/mBio.00543-17.
