# Supplementary material for: Virulence Determinants and Plasmid-Mediated Colistin Resistance mcr Genes in Gram-Negative Bacteria Isolated From Bovine Milk
Source: Front Cell Infect Microbiol. 2021 Nov 23;11:761417. doi: 10.3389/fcimb.2021.761417 (PMC8650641; doi:10.3389/fcimb.2021.761417)
Supplement: Supplementary file 5 [file Table_3.doc]

**Supplementary Table 3:** Agar well diffusion results and MICs of the most available disinfectants against some Gram-negative colistin-resistant bacteria

| **Disinfectant** | | **Bacteria** | | **Disinfectant concentration %** | | | | | | | | | | | | | | | | | | | | | **MIC**  **(%)** |
| --- | --- | --- | --- | --- | --- | --- | --- | --- | --- | --- | --- | --- | --- | --- | --- | --- | --- | --- | --- | --- | --- | --- | --- | --- | --- |
|  |  | | **10** | | **20** | | **30** | | **40** | | **50** | | **60** | | **70** | | **80** | | **90** | | **100** | |  |  | |
| Chlorhexidine gluconate 0.5% | | *E. coli* | | 11±0.3 | | 11±0.7 | | 14±1.1 | | 15±0.6 | | 24±0.7 | | 38±2.3 | | 38±3.6 | | 39±2.7 | | 39±3.6 | | 39±2.7 | | | 40 |
|  | | *K. pneumoniae* | | 12±0.4 | | 13±0.3 | | 13±1.2 | | 17±1.1 | | 30±2.1 | | 36±2.2 | | 36±2.6 | | 36±2.6 | | 37±2.4 | | 37±3.4 | | | 40 |
|  | | *P. aeruginosa* | | 27±1.3 | | 27±0.5 | | 28±0.9 | | 28±2.3 | | 28±1.5 | | 29±0.7 | | 30±2.8 | | 30±2.5 | | 30±2.5 | | 30±2.6 | | | 10 |
|  | | *A .hydrophila* | | 32±1.6 | | 32±0.5 | | 33±1.5 | | 35±3.1 | | 35±1.6 | | 35±1.5 | | 35±2.6 | | 36±2.5 | | 36±1.6 | | 36±3.1 | | | 10 |
| Iodine 0.5% | | *E. coli* | | 0 | | 0 | | 0 | | 0 | | 5±0.3 | | 8±0.8 | | 11±0.2 | | 15±0.7 | | 18±0.3 | | 20±1.4 | | | 80 |
|  | | *K. pneumoniae* | | 0 | | 2±0.1 | | 4±0.2 | | 5±0.3 | | 7±0.2 | | 10±0.2 | | 11±0.2 | | 16±1.3 | | 20±0.3 | | 22±2.3 | | | 80 |
|  | | *P. aeruginosa* | | 0 | | 0 | | 0 | | 0 | | 4±0.1 | | 5±0.1 | | 8±0.1 | | 10±0.2 | | 10±0.1 | | 10±0.2 | | | ND |
|  | | *A. hydrophila* | | 0 | | 0 | | 1±0.0 | | 3±0.2 | | 5±0.3 | | 7±0.1 | | 8±0.1 | | 11±0.4 | | 13±0.2 | | 14±1.3 | | | ND |
| Hydrogen peroxide 6% | | *E. coli* | | 8±0.4 | | 8±0.5 | | 10±0.3 | | 12±0.6 | | 16±0.8 | | 18±2.1 | | 19±1.3 | | 19±3.4 | | 20±1.4 | | 20±1.4 | | | 50 |
|  | | *K. pneumoniae* | | 35±2.7 | | 35±0.7 | | 36±1.2 | | 36±1.7 | | 37±1.6 | | 37±3.2 | | 37±2.6 | | 38±2.8 | | 39±3.7 | | 39±2.7 | | | 10 |
|  | | *P. aeruginosa* | | 28±0.9 | | 30±0.6 | | 31±2.1 | | 31±2.1 | | 32±1.5 | | 33±3.3 | | 36±2.3 | | 36±1.6 | | 37±2.8 | | 37±2.7 | | | 10 |
|  | | *A. hydrophila* | | 32±1.5 | | 32±0.5 | | 32±2.3 | | 35±1.9 | | 36±1.7 | | 36±2.9 | | 36±4.1 | | 38±2.7 | | 38±2.1 | | 40±3.8 | | | 10 |
| Ethanol 70% | | *E. coli* | | 0 | | 0 | | 0 | | 0 | | 0 | | 0 | | 0 | | 0 | | 0 | | 0 | | | ND |
|  | | *K. pneumoniae* | | 0 | | 0 | | 0 | | 0 | | 0 | | 0 | | 0 | | 0 | | 0 | | 0 | | | ND |
|  | | *P. aeruginosa* | | 0 | | 0 | | 0 | | 0 | | 0 | | 0 | | 0 | | 0 | | 0 | | 0 | | | ND |
|  | | *A. hydrophila* | | 0 | | 0 | | 0 | | 0 | | 0 | | 0 | | 0 | | 0 | | 0 | | 0 | | | ND |

ND, not detected; Bacterial isolates showed inhibition zones` diameters beyond 15 mm were considered sensitive.
